# HIV-1 enhances mTORC1 activity and repositions lysosomes to the periphery by co-opting Rag GTPases

**DOI:** 10.1038/s41598-017-05410-0

**Published:** 2017-07-14

**Authors:** Alessandro Cinti, Valerie Le Sage, Miroslav P. Milev, Fernando Valiente-Echeverría, Christina Crossie, Marie-Joelle Miron, Nelly Panté, Martin Olivier, Andrew J. Mouland

**Affiliations:** 1 HIV-1 RNA Trafficking Laboratory, Lady Davis Institute at the Jewish General Hospital, Montréal, Québec H3T 1E2 Canada; 2 Department of Medicine and the Division of Experimental Medicine, McGill University, Montréal, Québec H3A 0G4 Canada; 3 Department of Zoology, University of British Columbia, Vancouver, British Columbia V6T 1Z4 Canada; 4 Department of Microbiology and Immunology, McGill University, Montréal, Québec H3A 2B4 Canada; 5 Molecular and Cellular Virology Laboratory, Virology Program, Institute of Biomedical Sciences, Faculty of Medicine, Universidad de Chile, Independencia, 834100 Santiago Chile

## Abstract

HIV-1 co-opts several host machinery to generate a permissive environment for viral replication and transmission. In this work we reveal how HIV-1 impacts the host translation and intracellular vesicular trafficking machineries for protein synthesis and to impede the physiological late endosome/lysosome (LEL) trafficking in stressful conditions. First, HIV-1 enhances the activity of the master regulator of protein synthesis, the mammalian target of rapamycin (mTOR). Second, the virus commandeers mTOR-associated late endosome/lysosome (LEL) trafficking and counteracts metabolic and environmental stress-induced intracellular repositioning of LEL. We then show that the small Rag GTPases, RagA and RagB, are required for the HIV-1-mediated LEL repositioning that is likely mediated by interactions between the Rags and the viral proteins, Gag and Vif. siRNA-mediated depletion of RagA and RagB leads to a loss in mTOR association to LEL and to a blockade of viral particle assembly and release at the plasma membrane with a marked concomitant reduction in virus production. These results show that HIV-1 co-opts fundamental mechanisms that regulate LEL motility and positioning and support the notion that LEL positioning is critical for HIV-1 replication.

## Introduction

The mammalian target of rapamycin (mTOR) is a conserved serine/threonine kinase, a member of the phosphatidylinositol 3-kinase (PI3K) family and it exists within two functionally distinct multiprotein complexes, mTOR complex 1 (mTORC1) and 2 (mTORC2)^1^. Activation of mTORC1 occurs in response to growth factors and nutrients to control protein homeostasis via regulation of translation, autophagy and proteasomal degradation^1^. mTORC1 can also be activated by oxidative stress (e.g., by Arsenite (Ars) treatment)^2^, although Arsenite leads to the repression of the global cellular mRNA translation through the assembly of stress granules (SGs)^3^. SGs are sites of mRNA triage that contain non-translating mRNAs, self-associating proteins including the RNA-binding protein TIAR^4^ as well as mTOR, that transits between SGs and the cytosol to regulate translation during cellular stress^5, 6^.

The amino acid (aa)-induced activation of mTORC1 is directly mediated by a class of small GTPases, named the Ras-related GTP binding (Rag) proteins^7^. In the presence of aa, RagA and RagB are GTP-loaded and induce translocation of mTORC1 from the cytoplasm to late endosomal/lysosomal (LEL) surfaces, thus bringing the complex into close proximity to its direct activator, Rheb^7^. During viral egress, malleable pools of the HIV-1-structural protein Gag and vRNA also associate with LEL membranes^8, 9^ for trafficking to the cell surface for virus assembly. Experimental observations indicate that HIV-1 induces mTOR phosphorylation and downstream signalling in renal tubular cells, and that rapamycin, a potent and specific inhibitor of mTORC1, inhibits virus replication in HIV-1-infected patients^10^ and in other experimental systems acting at various levels of replication^11, 12^. Finally, RagA was previously found to associate with components of the LEL-associated HIV-1 ribonucleoprotein (RNP)^8, 13^ suggesting its involvement in viral RNA fate and metabolism.

In this study, we demonstrate that HIV-1 enhances mTORC1 activity in the presence of nutrients to favor its own replication. The synthesis of the HIV-1 structural protein Gag was abruptly downregulated upon pharmacological inhibition of mTOR but nevertheless, *de novo* synthesis of Gag proceeded and synthesis was partially restored. Gag synthesis also resisted the proteolytic targeting of mTOR suggesting a switch to non-canonical translation initiation. Furthermore, we show for the first time that HIV-1 commandeers lysosomal positioning in a RagA/RagB GTPase-dependent manner to maintain a peripheral cytoplasmic distribution of mTOR-associated lysosomes. Silencing the GTP-binding subunit of the Rag GTPase, RagA and RagB disrupted mTOR localization to the lysosome and inhibited HIV-1’s effects on lysosome positioning and trafficking. Depletion of the Rags also led to a marked decrease in virus production due to a block in virus budding and release. Altogether, these results indicate that HIV-1 hijacks the Rag GTPase/mTORC1 complex to modulate host cell function for optimal virus trafficking, assembly and/or budding.

## Results

### HIV-1 induces the mTORC1 activity

**Figure 1:**
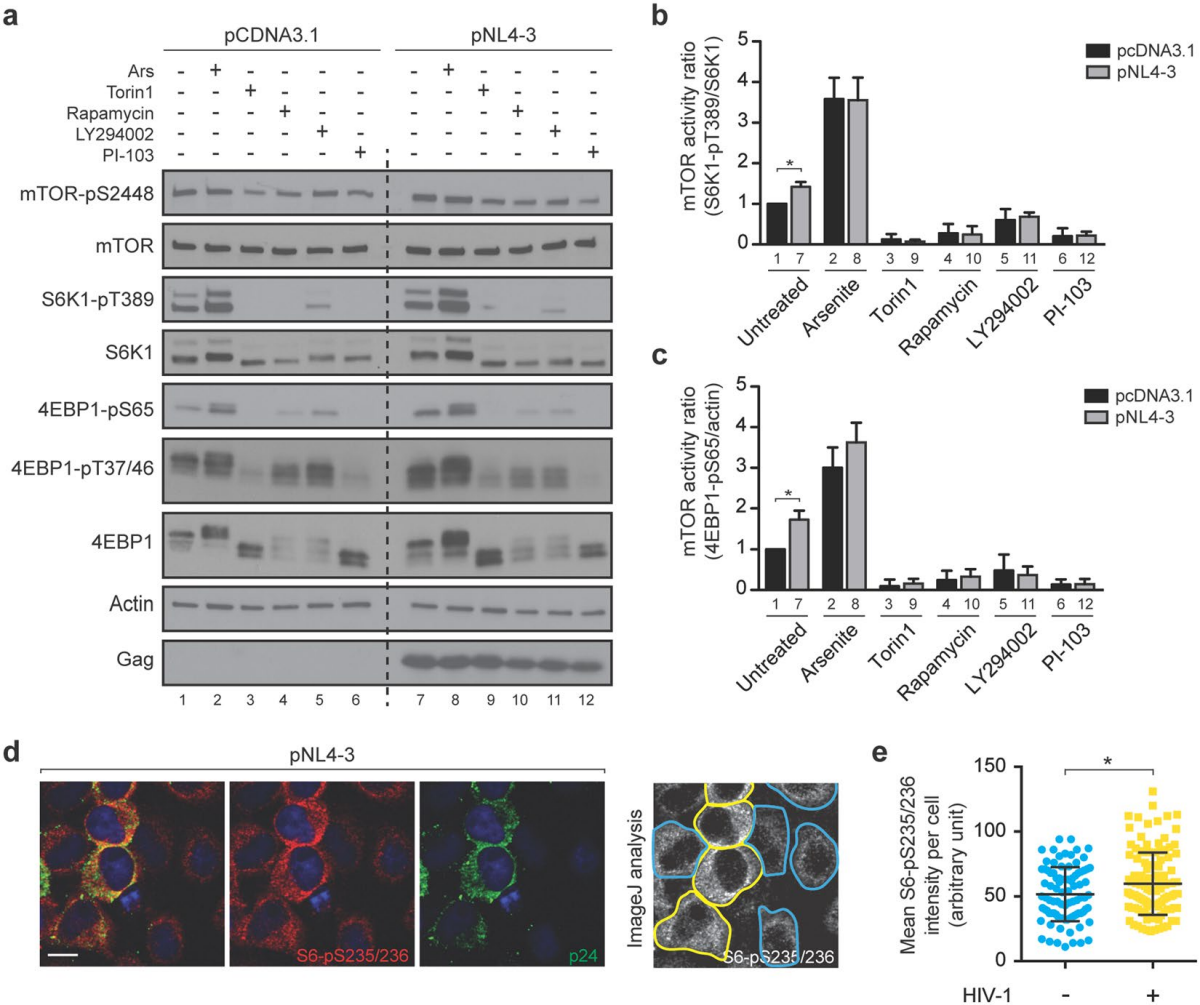
HIV-1 induces mTORC1 activity. (**a**) HeLa cells were transfected with either pcDNA3.1 or pNL4-3 for 24 h before incubation without or treated with 500 *μ*M Ars, 250 nM Torin1, 100 nM Rapamycin, 20 *μ*M LY294002 or 5 *μ*M PI-103 for 1 h. Cell lysates were subjected to SDS-PAGE, immunoblotted and probed with the indicated antibodies. Data shown are representative of three independent experiments. Full-length blots are shown in Supplementary Fig. 5. (**b**) The graph shows the ratio of S6K1-pT389/total S6K1 for mock or HIV-1-transfected cells from panel a. Values were normalized against untreated cells transfected with pcDNA3.1. The results are presented as the mean ± S.D. from three different experiments. P value is indicated by *(p < 0.05). (**c**) The graph shows the ratio of 4EBP1-pS56/actin for mock or HIV-1-transfected cells from panel a. Values were normalized against untreated cells transfected with pcDNA3.1. The results are presented as the mean ± S.D. from three different experiments. P value is indicated by *(p < 0.05). (**d**) Immunofluorescence was performed on HeLa cells transfected with pNL4-3. The mean fluorescence intensity of S6-pS235/236 was calculated by using ImageJ from at least 100 HIV-1-positive or HIV-1-negative cells. (**e**) The graph shows the mean S6-pS235/236 fluorescence intensity from three different experiments. P value is indicated by *(p < 0.05).

To determine the activation status of the mTORC1 pathway, lysates from HeLa cells mock-transfected with pcDNA3.1 or transfected with the infectious HIV-1 molecular clone pNL4-3 were prepared for Western blotting and probed for the expression of total and phospho forms of S6K1 and 4EBP1, which are considered to be robust readouts for mTORC1 activity. When compared to mock conditions, HIV-1-expressing cells exhibited significantly enhanced phosphorylated S6K1 (S6K1-pT389) and 4EBP1 (as judged by measuring 4EBP1-pS65) levels (Fig. 1a, lanes 1 and 7; Fig. 1b,c), indicating that HIV-1 enhances mTORC1 activity.

Rapamycin and Torin1 directly inhibit mTOR activity, while LY294002 and PI-103 are potent inhibitors of class I PI3Ks^14^, acting upstream of mTOR. Mock-transfected HeLa cells were treated with each of these mTOR inhibitors (Fig. 1a–c, lanes 3–6), resulting in decreased S6K1-pT389 and 4EBP1-pS65, as compared to untreated cells as expected. Conversely, oxidative stress, as induced by Ars, mediates the activation of mTORC1^2^. The incubation of mock or HIV-1-expressing cells with Ars caused an increase in S6K1-pT389 (Fig. 1a, lanes 2 and 8). HIV-1 was unable to cause a significant change in the activation status of mTOR and its downstream substrates in the presence of the four mTOR inhibitors listed above (Fig. 1b,c, lanes 9–12). To further confirm these results we assesed mTORC1 activity by immunofluorescence based on the phosphorylation status of the downstream marker, ribosomal S6 (S6-pS235/236) (Fig. 1d). This analysis showed that in HIV-1-positive cells, S6-pS235/236 levels were enhanced when compared to the intensity of the staining observed in HIV-1-negative cells, confirming that HIV-1 upregulated the mTORC1 activity (Fig. 1e). Overall, these data indicate that HIV-1 enhances mTOR activity at late stages of viral replication but is unable to maintain it upon pharmacological inhibition that affect mTOR and the mTORC1 pathway directly or indirectly.

### mTORC1 activity is required for optimal Gag synthesis

**Figure 2:**
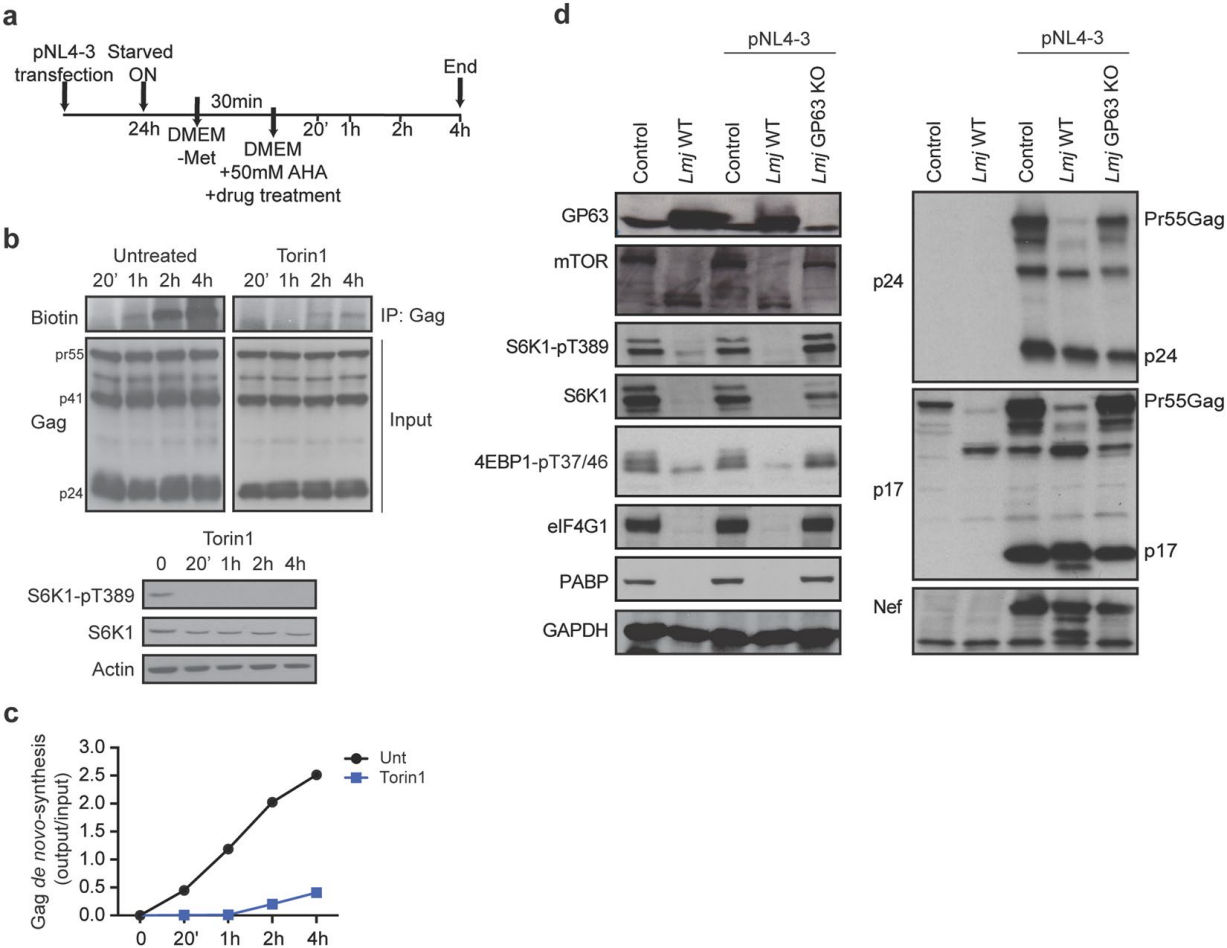
mTOR activity is required for optimal viral-protein synthesis. (**a**) Schematic of experimental protocol. (**b**) HeLa cells were transfected with pNL4-3, left untreated or treated with 250 nM Torin1 in medium containing AHA. At the indicated times, cell lysates were collected and Click-it chemistry was performed followed by immunoprecipitation with rabbit anti-p24 antibody. Membranes were probed with the indicated antibodies. (**c**) Densitometry quantification of de novo synthesized, biotinylated Gag from panel b was determined by ImageJ analysis. For each time point, the values presented in the graph are normalized against the total amount of Gag in the cell lysate (refer to Supplementary Fig. 1 for total protein synthesis). Full-length blots are shown in Supplementary Fig. 5. (**d**) HeLa cells were transfected with either pcDNA3.1 or pNL4-3 and infected with L. major (lmj) WT or GP63 KO. Cell lysates were subjected to SDS-PAGE, immunoblotted and probed with the indicated antibodies against host (left panels) and viral (right panels) antigens.

To determine whether mTOR activity is necessary for HIV-1 expression we examined *de novo* Gag protein synthesis upon treatment with Torin1, a highly specific, small molecule inhibitor that binds the mTOR kinase domain^15^. To quantify *de novo* Gag synthesis in untreated and Torin1-treated HeLa cells, we labeled newly synthesized proteins with L-azidohomoalanine (AHA), a substitute for methionine (Fig. 2a) and susequentially ligated the AHA-labeled proteins to biotin. Newly synthesized proteins can be then identified using an anti-biotin antibody. Synthesis of Gag was observed to increase over time in untreated and Torin1-treated HeLa cells (Fig. 2b,c). As expected, compared to untreated controls there was a marked reduction in Gag synthesis in the presence of Torin1 that recovered partially even in the presence of drug in a situation when both translation (Fig. 2b,c) and total protein synthesis are blocked (Supplementary Fig. 1a,b), supporting the notion that HIV-1 overcomes the translational block due to mTOR inhibition. Considering that accumulation of Gag under these conditions might be consequence of increased stability of either Gag-RNA or Gag-protein, we investigated if Torin1 treatment induced accumulation of the two. Consistent with our hypothesis that HIV-1 can overcome the inhibition of cap-dependent mRNA translation, we did not observe any change in Gag protein or RNA levels induced by Torin1 in cells treated with translation or transcription repressors (Supplementary Fig. 1c,d).

Next, we explored how HIV-1 overcomes the blockade to translation imposed by the cleavage of mTOR. We therefore co-infected HIV-1-expressing cells with the parasite *Leishmania major* because it subverts the translation machinery by completely cleaving mTOR via the activity of the parasite’s surface protease, GP63^16^. *L. major* infection also inactivates the eIF4F complex and cap-dependent translation^17^. HIV-1-transfected HeLa cells were infected with *L. major* wild-type (WT) or GP63 knockout (KO) and the cleavage status of proteins involved in the mTORC1 pathway was investigated. *L. major* WT led to the cleavage of mTOR, S6K1, eIF4G and PABP with concomitant hypophosphorylation of 4EBP1, whereas the GP63 KO did not cleave (Fig. 2d, left panels). Under these conditions, host translation initiation and viral protein synthesis were reduced (Fig. 2d, right panels). Quantification of Gag-specific bands from the p24 and p17 blots illustrated that Gag expression was reduced by approximately 40% in cells infected with *L. major* WT as compared to control cells. Taken together, these results indicate that the protein synthesis repression that is imposed by the inhibition of cap-dependent mRNA translation can be overcome by HIV-1, while mTORC1 activity appears to be required for optimal Gag expression.

### HIV-1 maintains cytoplasmic positioning of LEL but not mTORC1 activation during nutrient deprivation

**Figure 3:**
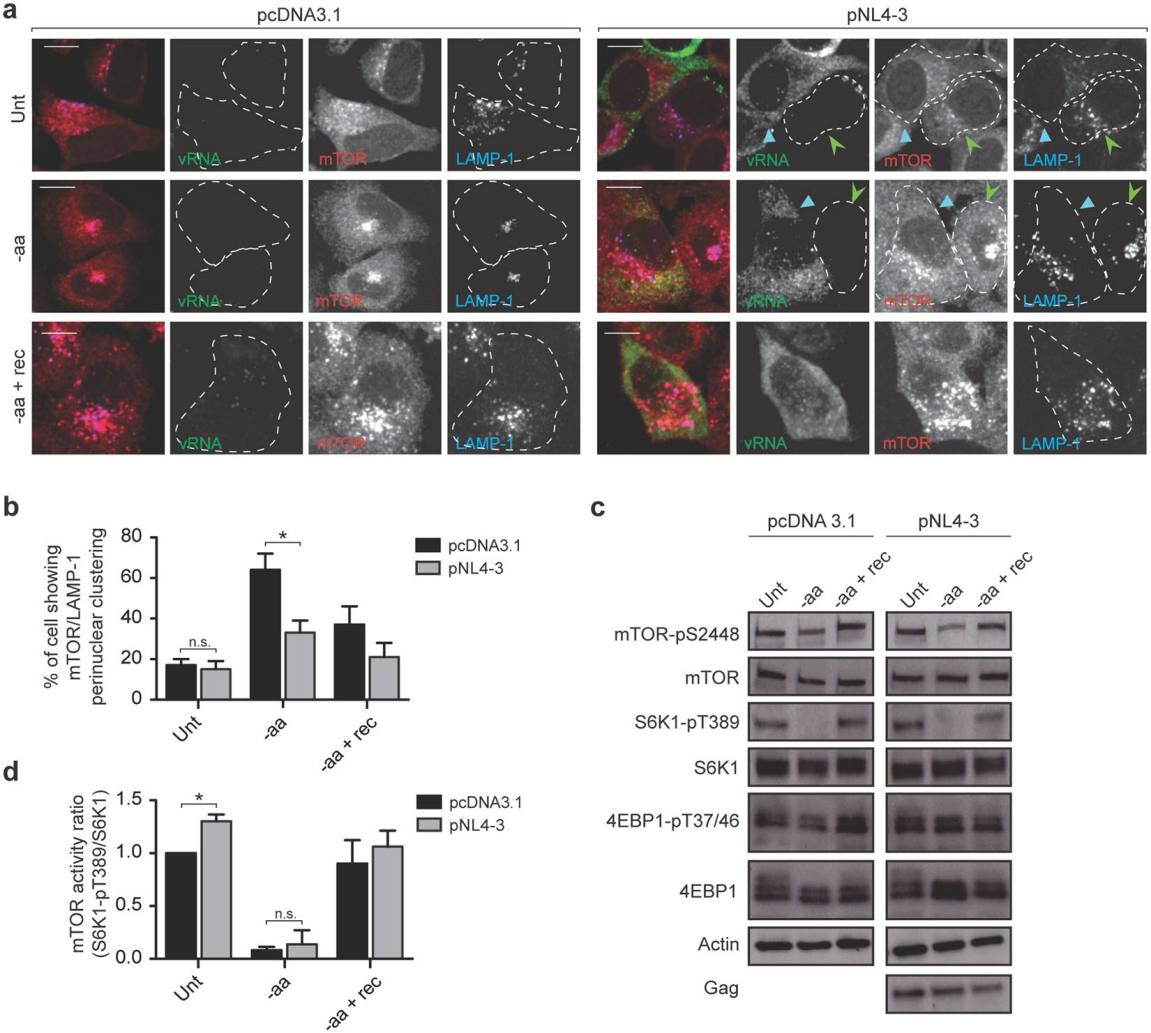
HIV-1 does not maintain mTORC1 activation during nutrient deprivation. (**a**) HeLa cells were transfected with either pcDNA3.1 or pNL4-3 and starved for amino acids (aa) (and serum) (1 h) and subsequently recovered with complete media (serum free) for an additional 30 minutes. HIV-1-expressing cells were identified by viral RNA (vRNA) using fluorescence *in situ* hybridization (FISH) and were stained with antibodies against the lysosomal population of mTOR (Cell Signaling 7C10) and LAMP-1. Light-blue arrowheads identify HIV-1-expressing cells, while HIV-1-negative cells in the same field are indicated with green arrowheads. A dashed line contours individual cells. Scale bars are 10 μm. (**b**) The percentage of mock or HIV-1-transfected cells showing perinuclear clustering of mTOR/LAMP-1 from panel a. The results are presented as the mean ± S.D. from three different experiments. P value is indicated by *(p < 0.05). (**c**) HeLa cells were transfected with either pcDNA3.1 or pNL4-3 and starved for amino acids (aa) (1 h) and subsequently recovered with complete media for an additional 30 minutes. Cell lysates were subjected to SDS-PAGE, immunoblotted and probed with the indicated antibodies. Full-length blots are shown in Supplementary Fig. 5. (**d**) The graphs show the ratio of S6K1-pT389/total S6K1 for mock or HIV-1-transfected cells as in panel c. Values were normalized against untreated cells transfected with pcDNA3.1. The results are presented as the mean ± S.D. from three different experiments. P value is indicated by *(p < 0.05); n.s.: no signifcant difference between the means.

Physiological starvation conditions have been shown to inhibit mTORC1 activity but to retain mTOR on lysosomes, which accumulate at juxtanuclear regions^18^. To determine whether the expression of HIV-1 affects mTORC1 localization upon aa starvation, HeLa cells were incubated in aa-free medium in the absence or presence of HIV-1 for 1 h and subsequently recovered with complete media for an additional 30 minutes. As shown in Fig. 3a, aa depletion induced an intracellular redistribution of mTOR and LAMP-1 to a juxtanuclear region in 64 ± 8% of mock-transfected cells. HIV-1-expressing cells, however, displayed juxtanuclear clustering of mTOR and LAMP-1 in only 33 ± 6% cells, indicating that HIV-1 imposes a blockade to starvation-induced lysosomal trafficking by dispersing LEL throughout the cytoplasm (Fig. 3a,b). Replenishment of aa resulted in a repositioning of mTOR and LAMP-1 to the periphery in both mock and HIV-1-transfected cells (Fig. 3a,b). The pronounced differences in the intracellular distribution of mTOR were not reflected in changes in its activation status, as shown in Fig. 3c,d.

Growth factors, amino acids and glucose are simultaneously required to maintain mTORC1 activation^19^. During brief aa (Fig. 3c,d) or glucose (Supplementary Fig. 2a,b) starvation, the levels of S6K1-pT389 and phosphorylated mTOR (mTOR-pS2448) decreased as compared to cells incubated in complete medium, regardless of the presence or absence of HIV-1. These data indicate that, although HIV-1 maintains a peripheral cytoplasmic distribution of mTOR-associated LEL, the virus is unable to alter mTORC1 signalling under nutrient deprivation.

### HIV-1 redistributes perinuclear clusters of mTOR-associated lysosomes

**Figure 4:**
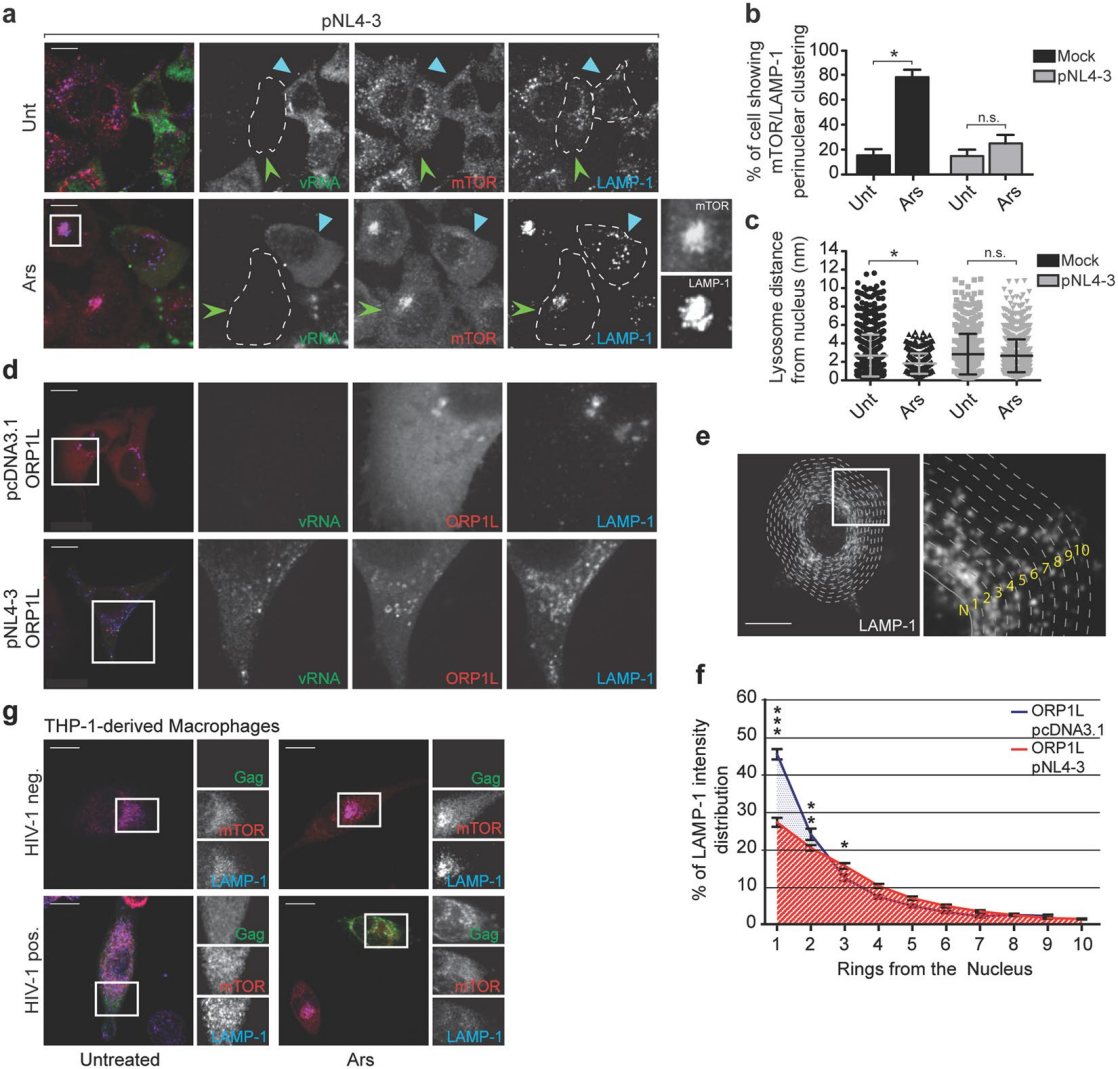
HIV-1 redistributes LEL. (**a**) HeLa cells were transfected with either pcDNA3.1 (not shown) or pNL4-3 and left untreated of stressed with 500 µM Ars. HIV-1-expressing cells were identified using fluorescence *in situ* hybridization (FISH) to localize vRNA and stained using antibodies against mTOR (Cell Signaling 7C10) and LAMP-1. Light blue arrowheads identify HIV-1-expressing cells, while HIV-1-negative cells in the same field are indicated with green arrowheads. A dashed line contours individual cells. Scale bars are 10 *μ*m. (**b**) The percentage of mock or HIV-1-transfected cells showing perinuclear clustering of mTOR/LAMP-1 from panel a. The results are presented as the mean ± S.D. from three different experiments. P value is indicated by *(p < 0.05); n.s.: no signifcant difference between the means. (**c**) The distance of lysosomes from the nucleus was quantified from at least 60 cells using the Spots Tool in Imaris software. The results are presented as the mean ± S.D. from three different experiments. P value is indicated by *(p < 0.05). (**d**) HeLa cells were co-transfected with either pcDNA3.1 or pNL4-3 and mRFP-ORP1L. HIV-1-expressing cells were identified using FISH to localize vRNA and stained for LAMP-1. Scale bar is 2 μm. (**e**) Diagram of the quantification analysis of the immunofluorescence intensity of LAMP-1 in concentric rings from the nucleus to the plasma membrane. Representative image from a pNL4-3 and mRFP-ORP1L transfected cell. (**f**) Quantitation of LAMP-1 immunofluorescence intensity in concentric rings spanning out from the nucleus in cells overexpressing mRFP-ORP1L co-transfected with either pcDNA3.1 or pNL4-3 (n = 40). Statistical significance was tested using a two-way ANOVA and P values are indicated by *(p < 0.05), **(p < 0.01) and ***(p < 0.001). (**g**) HIV-1-expression was induced by doxycycline (2 μg/mL) in an inducible THP-1-derived macrophage cell line for 72 h. Cells were left untreated or stressed with Ars for 1 h before fixation. HIV-1-expressing cells were identified by Gag-GFP expression and stained for mTOR (Cell Signaling 7C10) and LAMP-1.

In response to oxidative stress (induced by Ars) or heat stress, mTOR is recruited and sequestered to stress granules (SGs) in an inactive state^5, 6^. Using an antibody that stains the SG-associated subpopulation of mTOR (as demonstrated in ref. 6), we detected mTOR in Ars-induced SGs, except in cells expressing HIV-1, as expected^20, 21^ (Supplementary Fig. 3a). Ars treatment also induces the juxtanuclear clustering of the LEL-associated subpopulation of mTOR that is uniquely identified with another immune antiserum (characterized in refs 22, 23) (Supplementary Fig. 3b). To assess the localization of mTOR under oxidative stress, HeLa cells were transfected with pcDNA3.1 or pNL4-3 and stressed with Ars, which induces translational arrest^24^. The lysosomal-associated subpopulation of mTOR accumulated in tight clusters in 78 ± 6% of non-transfected cells within 1.8 ± 1 μm from the nucleus as compared to 18 ± 3% with an average distance from the nucleus of 2.6 ± 2.2 μm in untreated cells (Fig. 4a–c), while SG-associated mTOR as well as the mTORC1-specific component Raptor were excluded from these clusters (Supplementary Fig. 3b,c). During oxidative stress induced by Arsenite in fact, as expected^6^, Raptor was recruited to SGs and was concomintantly released from its lysosomal localization (Supplementary Fig. 3c), confirming that at least two different sub-populations of mTOR can be distinguished under oxidative conditions.

Strikingly, juxtanuclear clustering was only apparent in 26 ± 7% of HIV-1-expressing cells treated with Ars (Fig. 4a,b) and instead, maintained a distribution of mTOR-LAMP-1 that was largely dispersed at distal sites of the cell with an average distance of 2.6 ± 1.7 μm from the nucleus, similarly to that found in in untreated cells (2.8 ± 2.1 μm; Fig. 4c). To gain further insight into the development of the juxtanuclear clustering of the LEL, we decided to analyse the dynamics of LEL trafficking in live cells during Arsenite treatment. For this purpose, we co-transfected LAMP1-GFP and pNL4-3/GagmRFP and 24 h later treated with Ars and examined the cells by live cell microscopy (LCM). LEL clustering was induced in control (HIV-1 negative) cells, but was impeded in pNL4-3/GagmRFP, HIV-1-expressing cells (Supplementary Movie 1).

Because HIV-1 influenced mTOR-LAMP-1 positioning in cells, we investigated how HIV-1 would influence LEL clustering when the oxysterol binding protein-related protein 1 Long (ORP1L) was overexpressed. ORP1L is a member of the Rab7-interacting lysosomal protein (RILP)/Rab7 complex that regulates LEL positioning by mediating the dissociation of dynein in a cholesterol-dependent manner^25, 26^. However the overexpression of ORP1L forces the perinuclear clustering of LEL^25^. In this experiment, HeLa cells were co-transfected with mRFP-ORP1L and pcDNA3.1 or pNL4-3 and LAMP-1 and vRNA were identified by immunofluorescence and FISH co-analyses (Fig. 4d). The LEL distribution was quantified using MetXpress software (Molecular Devices) by measuring the intensity of LAMP-1 staining in concentric rings around the nucleus (Fig. 4e). The intensity of LAMP-1 staining within each ring was quantified against the total intensity of the cell to determine the LEL displacement (Fig. 4f). Overexpression of ORP1L resulted in 45 ± 1.3% of LEL located within the first concentric ring, whereas, HIV-1 mediated a reduction to 27 ± 1.2% in LEL in the first with a statistically significant increase in LAMP-1 staining in the distal ring 3 (Fig. 4f). These findings confirm the ability of HIV-1 to promote LEL positioning at the cellular periphery, counteracting their centripetal movement induced by different type of stimuli.

We next examined whether HIV-1 alone mediated the repositioning of LEL in monocyte-derived macrophage THP-1 cells, a primary HIV-1 target cell^27^. Ars treatment again led to juxtanuclear LAMP-1 positioning in HIV-1-negative cells (Fig. 4g), while mTOR and LEL in HIV-1-expressing macrophages were widely distributed throughout the cytoplasm, regardless of the addition of Ars (Fig. 4g). These data indicate that HIV-1 overcomes the juxtanuclear LEL positioning imposed by Ars, suggesting that the virus affects mTORC1 distribution by maintaining the dispersion of LEL throughout the cytoplasm. Taken together, these data demonstrate that HIV-1 commandeers LEL trafficking, and as a consequence the mTOR positioning, toward the periphery in the face of forced redistribution to juxtanuclear positions.

### Rag GTPases are necessary for HIV-1 to maintain peripheral LEL distribution and for its optimal budding and release

**Figure 5:**
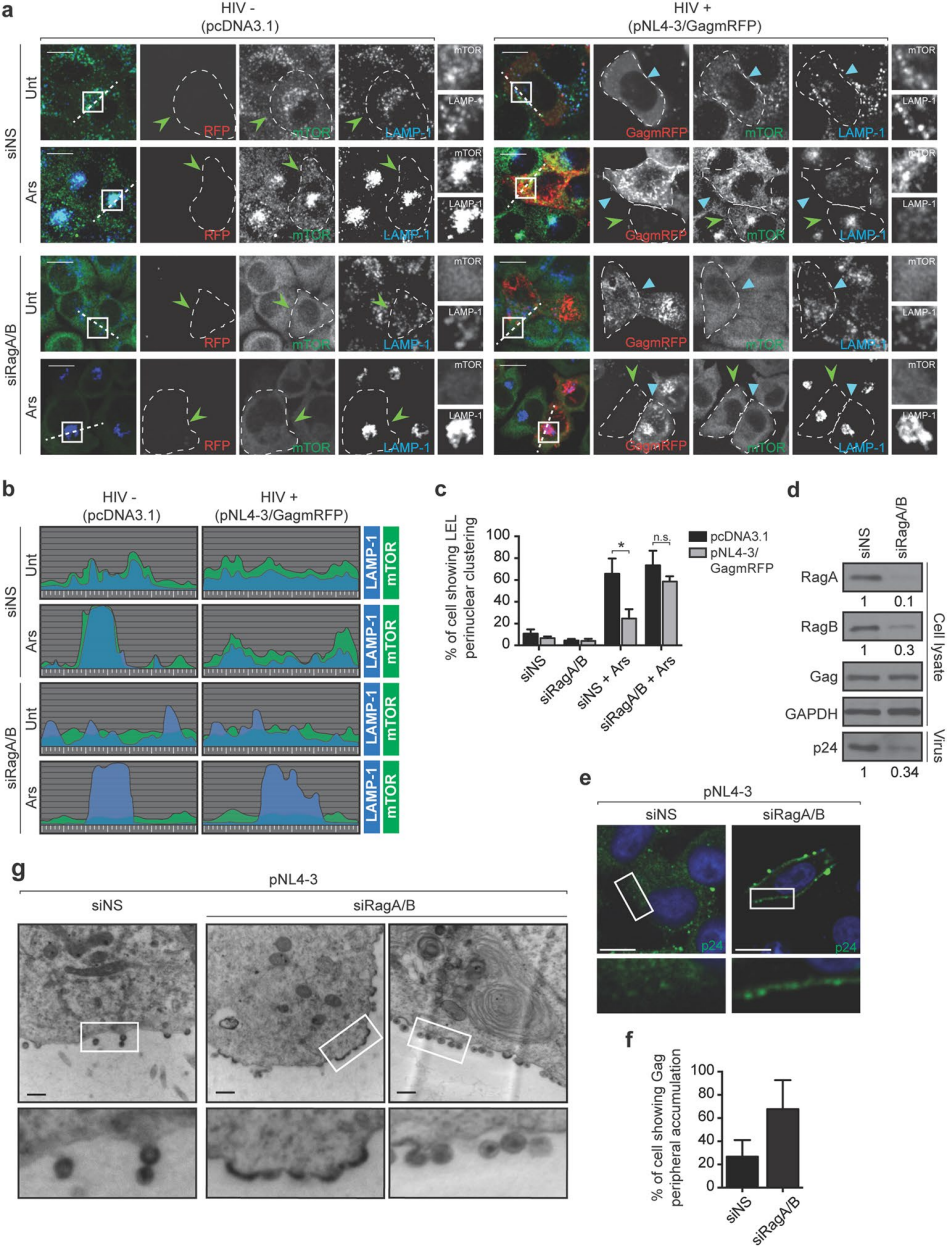
RagA and RagB are necessary for HIV-1 to control LEL trafficking and for the viral budding and release. (**a**) HeLa cells, treated with non-silencing (NS) siRNA or siRNAs targeting RagA and RagB mRNAs, were transfected with empty vector control, pCDNA3.1 or HIV-1 using pNL4-3/GagmRFP, and then mock treated or treated with Ars. Cells were stained for mTOR (Cell Signaling 7C10) and LAMP-1. Light blue arrowheads identify HIV-1-expressing cells, while HIV-1-negative cells are indicated with green arrowheads. A dashed line contours individual cells. Scale bars are 10 *μ*m. (**b**) The graphs represent the fluorescence intensities of mTOR (green) and LAMP-1 (blue) across the dashed lines from panel a. (**c**) The percentage of mock or HIV-1-transfected cells showing perinuclear clustering of LEL from panel a. The results are presented as the mean ± S.D. from three different experiments. P value is indicated by *(p < 0.05); n.s.: no signifcant difference between the means. (**d**) HeLa cells were co-transfected with the HIV-1 pNL4-3 and either non-sense (NS) siRNA or siRNA against RagA and RagB. HIV-1 particles were collected from the supernatant after filtration and ultracentrifugation. Cell lysates and virus particles were separated by SDS-PAGE, transferred to nitrocellulose and probed with the indicated antibodies. siRNA-mediated fold changes in RagA, RagB and virus production are indicated below insets in panel d in this representative experiment. Full-length blots are shown in Supplementary Fig. 5. (**e**) HeLa cells were co-transfected with the HIV-1 pNL4-3 and either non-sense (NS) siRNA or siRNA against RagA and RagB. Cells were fixed 24 h after transfection and stained for p24. (**f**) The percentage of HIV-1 and siRagA/B transfected cells showing peripheral accumulation of Gag from e. The results are presented as the mean ± S.D. from three different experiments. (**g**) HeLa cells were transfected as in e and subjected to transmission electron microscopy.

RagA and RagB recruit mTORC1 to lysosomal membranes and regulate its activity during aa starvation^7^. RagA and RagB knockdown impaired mTORC1 activity, as indicated by a reduction in S6K1-pT389 to a similar extent both in mock- and HIV-1-expressing cells (Supplementary Fig. 4a,b). In an attempt to delineate the mechanism of HIV-1 lysosomal redistribution and knowing that RagA was previously identified to co-immunoprecipitate in Staufen1-containing HIV-1 RNPs^13^, we depleted RagA and RagB by siRNA (70–90% depletion, Fig. 5d). As expected, silencing of RagA and RagB resulted in a diffuse localization of mTOR throughout the cytoplasm in both pcDNA3.1 and pNL4-3/GagmRFP transfected cells, as compared to the non-silencing siRNA (siNS) control in which mTOR colocalized with LAMP-1 (Fig. 5a,b). In HIV-1-expressing cells, Ars treatment caused a juxtanuclear accumulation of LAMP-1 in 24 ± 6% of siNS control cells (Fig. 5a,c), whereas, in striking contrast, 59 ± 6% of cells knocked down for RagA and RagB displayed a juxtanuclear accumulation similar to that observed in the HIV-1-negative cells (Fig. 5a,c). These results indicate that HIV-1 requires RagA and/or RagB to block the centripetal (inbound) repositioning of LAMP-1-positive LEL following Ars treatment.

RagA and B silencing has been shown to be detrimental to Andes virus replication^28^. To determine the importance of RagA and RagB in HIV-1 replication, we first examined virus production upon depletion of RagA and RagB. Interestingly, we found that siRNA-mediated depletion of RagA and RagB resulted in an 62 ± 17% (n = 3) decrease in HIV-1 virus production as determined by western blotting (Fig. 5d). Considering that the HIV-1 structural protein, Gag and the viral genomic RNA and several host proteins co-traffic on LEL during viral egress^8, 9, 29, 30^, we performed laser scanning confocal as well as transmission electron microscopy (EM) analyses on HeLa cells expressing HIV-1 and depleted for RagA and RagB. Laser scanning confocal analysis revealed that, in contrast to control cells transfected with siNS, cells depleted for RagA and RagB exhibited an increased accumulation of Gag at the plasma membrane (Fig. 5e,f). Strikingly, these results were confirmed by electron microscopy such that virus assembly in RagA and RagB-depleted cells was either incomplete exhibited by electron dense accumulations of virions at the cell surface (Fig. 5g) or completed, but virus particles were unable to pinch off the outside surface of the membrane (Fig. 5g). Taken together, our gene silencing studies, viral production experiments as well as microscopy analysis, demonstrate for the first time that the small GTPases are important for HIV-1 to control LEL trafficking and for the viral assembly and/or release.

### Gag and Vif interact with RagA and RagB

**Figure 6:**
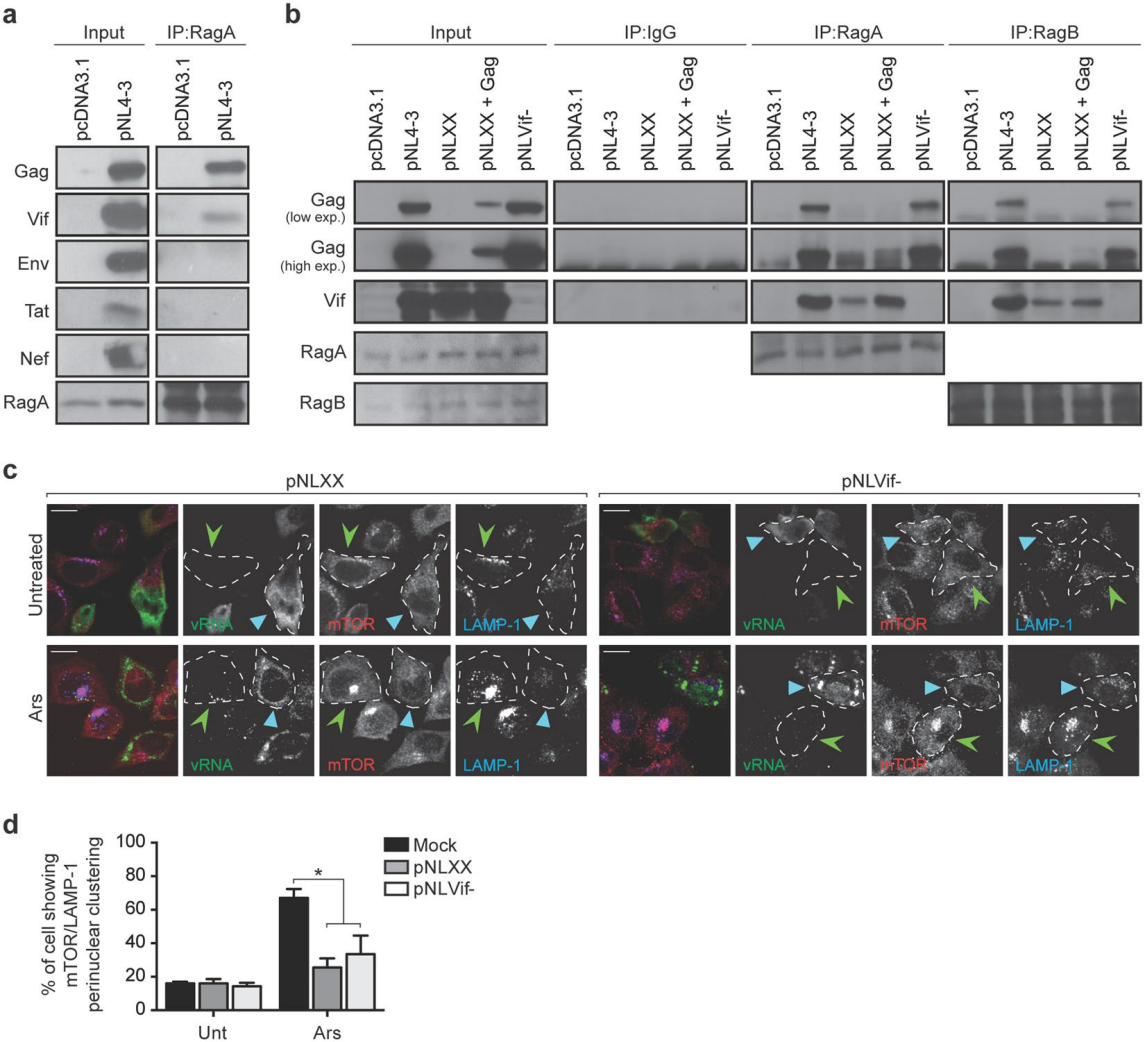
RagA and RagB interact with Gag and Vif. (**a**) Lysates from control pcDNA3.1- or pNL4-3-transfected HeLa cells were subjected to immunoprecipitation with RagA antibodies. Samples were subjected to SDS-PAGE followed by transfer to nitrocellulose membranes and probed with the indicated antibodies. Full-length blots are shown in Supplementary Fig. 5. (**b**) Lysate from pcDNA3.1-, pNL4-3-, pNLXX−, pNLXX+ pSVGag− and pNLVif–transfected HeLa cells were subjected to immunoprecipitation with RagA and RagB antibodies or isotype rabbit IgG control. The blots were probed with anti-p24, anti-Vif, anti-RagA and anti-RagB antibodies. Full-length blots are shown in Supplementary Fig. 5. (**c**) HeLa cells were transfected with either pNLXX or pNLVif- and left untreated (Unt) or stressed with 500 µM Ars for 1 h. HIV-1-expressing cells were identified using FISH to localize vRNA and stained using antibodies against mTOR (Cell Signaling) and LAMP-1. Light blue arrowheads identify pNLXX or pNLVif- expressing cells, while pNLXX or pNLVif- negative cells in the same field are indicated with green arrowheads. A dashed line contours individual cells. Scale bars are 10 μm. (**d**) The percentage of mock, pNLXX or pNLVif- transfected cells showing perinuclear clustering of mTOR/LAMP-1 from panel c. The results are presented as the mean ± S.D. from three different experiments. P value is indicated by *(p < 0.05).

As a previous study identified RagA as being associated with the HIV-1 RNP^13^, we immunoprecipitated RagA to determine which HIV-1 proteins are capable of interacting and potentially responsible for redistributing mTOR-associated lysosomes. Western blotting with several anti-HIV-1 antibodies showed that Gag and Vif were specifically captured by RagA, while Env (gp120), Tat and Nef were not pulled down (Fig. 6a). To confirm the specificity of these interactions, as RagA and RagB share 98% identity, we performed immunoprecipitations with antibodies against RagA, RagB or an IgG isotype control using HeLa cell lysates transfected with pcDNA3.1, pNL4-3, pNLXX (lacks Gag expression) or pNLVif- (lacks Vif expression). Surprisingly, co-immunoprecipitation of Vif with RagA (and partially with RagB) was reliant on Gag expression as the amount of Vif pulled down decreased in the absence of Gag (pNLXX) but was restored upon Gag (pSVGag) expression *in trans* (Fig. 6b). The pull down of Gag together with RagA and RagB was independent of Vif as equal amount of Gag were co-immunoprecipitated in pNL4-3 and pNLVif- (Fig. 6b).

To address whether the absence of Gag or Vif in the context of HIV-1 affected lysosomal positioning in the presence of Ars, HeLa cells were transfected with pNLXX or pNLVif-. A dispersed intracellular distribution of LAMP-1-positive LEL was observed in those cells expressing HIV-1 viruses that lacked either Gag (25 ± 6% cells showing clustering) or Vif (35 ± 8% cells showing clustering) upon treatment with Ars (Fig. 6c,d) as compared to that observed in the mock-transfected cells (68 ± 5% cells showing clustering) (Fig. 6d). Taken together, the data suggest that although Gag and Vif co-immunoprecipitate with RagA and RagB, the absence of one is not sufficient to abolish HIV-1 dispersal of LAMP-1-positive vesicles.

## Discussion

In this study, we report that HIV-1 is able to modulate the activity of mTORC1, which appears to be necessary for the optimal expression of the structural viral protein Gag. Nevertheless HIV-1 is not able to sustain the activity of mTOR in conditions of nutrient deprivation or when mTOR is pharmacologically inhibited. Furthermore, we demonstrate that HIV-1 is able to redistribute mTOR-associated LEL that typically accumulate at a juxtanuclear localization in the presence of Ars or as consequence of nutrient starvation, an activity that we demonstrated for the first time to be dependent on the small GTPases RagA and RagB. A specific interaction between RagA/RagB and Gag/Vif was detected by co-immunoprecipitation, but neither of the two viral proteins was essential to block LEL clustering in the presence of Ars. Depletion of RagA and RagB reduced virus production and caused accumulation of viral particles at the plasma membrane, indicating that they are necessary for efficient virus assembly and/or release.

mTOR is essential for controlling mRNA translation, growth, cell cycling and autophagy^1^, thus maintaining its activation during infection is an important viral evasion mechanism of the host stress response. In dendritic cells, the HIV-1 glycoprotein Env rapidly induces mTOR signalling upon infection^31^. As mTOR partitions to SGs when translation is inhibited^6^, HIV-1’s ability to inhibit SG assembly via at least two distinct mechanisms could also ensure the availability of mTOR for exploitation by the virus^20, 21^. Under physiological conditions our results show a modest, yet significantly enhanced activity of mTORC1 in HIV-1-expressing cells (Fig. 1), indicating that sustaining mTORC1 activation is important for HIV-1. However, HIV-1 can not maintain mTORC1 activity (as assessed by S6K1 phosphorylation status) after pharmacologic treatment with drugs that block mTOR directly (Torin1 and rapamycin) or those that block mTOR indirectly (LY294002 and PI-103)^14^, and when aa or glucose is depleted from the medium. These results suggest that HIV-1 targets mTORC1 at a yet undefined point of the mTORC1 pathway. Torin1 treatment was recently shown to lower HIV-1 production in CD4 + T lymphocytes^32^, a result that agrees with the rapid, acute decline in Gag expression we observe (Fig. 2b). Nevertheless, HIV-1 expression can be rescued in an mTOR-independent manner, as evidenced by a recovery of Gag synthesis during Torin1 treatment or even when mTOR is completely cleaved in *L. major* infection, although GP63 is quite pleiotropic in its proteolytic activity (Fig. 2). The *L. major* GP63, in fact, cleaves the mTOR complex to downregulate the host translational machinery by activating the transcriptional repressor 4EBP1. Under these conditions viral protein synthesis was reduced by approximately 40% in cells infected with *L. major* WT. Since mTORC1 activation directly controls cap-dependent translation, it is reasonable to speculate that, while mTORC1 activity appears to be required for optimal viral proteins expression, Gag may also accumulate due to genomic RNA translation initiation via the IRES^33, 34^. Consistently, cells appear able to increase IRES-dependent mRNA translation, when cap-dependent initiation is reduced by rapamycin^35^. While mTOR inhibitors such as rapamycin, INK128 and Torin1 have been shown to exert anti-HIV-1 activity at several steps of the replication cycle^11, 32, 36, 37^, their effects on different components of the immune system, for example on regulatory T (T_Reg_) cells, could also contribute to their anti-viral role^38^. For these reasons, defining the mechanisms by which mTOR inhibition affects HIV-1 replication will require further work. Interestingly, very recent findings posed a novel functional link between mTOR activity and HIV-1 latency^39, 40^, highlighting once more the importance of enhancing our understanding of the relationship between mTOR and HIV-1.

Our novel finding that HIV-1 hijacks LEL positioning in conditions when these vesicles are directed towards juxtanuclear regions (during aa starvation, arsenite treatment or when ORP1L is overexpressed) reveals another way in which HIV-1 commandeers major host cell machineries. HIV-1 has been shown to promote distal positioning of cholesterol-rich membranes^41, 42^ that serve as cargo for the HIV-1 RNPs that are composed of Gag, the genomic RNA as well as several host proteins^8, 9, 30^. Moreover, recent work has unveiled additional roles for repositioning Rab7a-positive late endosomes to distal sites for HIV-1 to counter the activity of host restriction factors^43^, as well as for targeting phospholipid kinases via Rab27a-laden late endosomes to generate scaffolds for Gag assembly at the plasma membrane^29^. For that, the modulation of endolysosomal pathways has been recently argued to be a strategic target to combat HIV-1 infection^44^.

Our results provide the first evidence that HIV-1 maintains the cytoplasmic positioning of LEL via the co-opting of the small GTPases, RagA and RagB. Nevertheless the two Rags appear to be essential to reach the optimal rate of viral assembly and budding. In our efforts to define the mechanism by which the Rags act, we show here for the first time that HIV-1 Gag and Vif interact with RagA and RagB. The interaction between Vif and RagA/RagB appears to depend on Gag synthesis, suggesting that the Gag/Vif-Rag(A/B) interaction might be promoted and/or stabilized to coincide with the late step of the replication cycle when Gag is abundantly expressed. Earlier work showed that several domains of Vif contribute to the interaction with Gag and that the interaction is critical for Vif function^45^. It is interesting to speculate on a novel role for Vif in vesicular trafficking that is independent from its activity against APOBEC3G^46^. It is also possible that, instead of a direct virus-host interaction between Gag/Vif-Rag(A/B), HIV-1 may co-opt the activity of RagA/RagB to redistribute LEL. Indeed recent findings have demonstrated that, in microglia and in mouse embryonic fibroblasts, Rag-Ragulator can function independently of mTOR signaling to modulate lysosome size and degradative capacity^47, 48^. The fact that the Rag GTPase can operate in an aa-independent fashion unrelated to mTORC1 could also explain why during nutrient deprivation the virus coordinates lysosomal positioning without affecting mTORC1 activity.

The repositioning of lysosomes has major impacts on their function^18, 26^, their intraluminal pH^49^ and their ability to recruit resident host proteins^50^. In particular, this can impact on mTOR activity^18^, as well as on viral proteins and RNP recruitment^8, 51^. A more peripheral positioning of lysosomes, in fact, could promote membrane fusion events with other endosome populations at the plasma membrane, or enhance the proximity between mTOR and upstream signalling molecules^18^ (even if discrepancies exists in this respect^52^). While the marker protein LAMP-1 co-traffics on LEL and coincides with HIV-1 assembly sites at the plasma membrane^8, 29^ topologically, these observations would be consistent with eventual LEL-plasma membrane fusion to allow for coupled viral protein synthesis and Gag assembly at the inner plasma membrane.

Typically, RNA viruses will usurp the mTORC1 pathway by modulating the phosphorylation of downstream effectors, S6K1 or 4EBP1^53^. In this manuscript we show that HIV-1 co-opts host vesicular trafficking and mTORC1 activation independently. Whether HIV-1-mediated LEL repositioning directly influences mTOR activity, as in the case of Andes virus^28^, or provides a means to target critical host and viral components to engage in viral protein synthesis and assembly remains to be fully defined.

## Materials and Methods

### Plasmids

pNL4-3 was obtained from NIH AIDS Reference and Reagent Program (ARRP). pNLVif- was provided by Dr. Klaus Strebel (NIH)^54^. pSVGag was a generous gift from Dr. Jaisri Lingappa (University of Washington)^55^. pNLXX is proviral clone that contains a six-nucleotide mutation and that produces an amber nonsense codon (TAG) in place of the gag initiation codon and a nonsense mutation within CA (residue 109, residue 241 of Pr55Gag) was provided by Dr. David Ott (NCI Frederick, MD)^56^. The mRFP-ORP1L construct was a generous gift from Dr. Jacques Neefjes (National Cancer Institute, Amsterdam, EU)^57^. pNL4-3/GagmRFP was provided by Dr. Wei-Shau Hu (NCI Frederick, MD). pcDNA3.1 was purchased from Invitrogen.

### Antibodies

Mouse anti-p24, mouse anti-Vif, rabbit anti-Nef, mouse anti-gp120 and mouse anti-Tat antibodies were obtained from NIH AIDS ARRP; rabbit anti-mTOR (sc-1549-R) and goat anti-TIAR antibodies were purchased from Santa Cruz Biotechnology; rabbit anti-RagA, rabbit anti-RagB, rabbit anti-mTOR (7C10), rabbit anti-S6K1, rabbit anti-4EBP1, rabbit anti-phospho-mTOR (Ser2448), rabbit anti-phospho-S6K1 (Thr389), rabbit anti-phospho-4EBP1 (Th37/46), rabbit anti-phospho-4EBP1 (Ser65) and rabbit anti-phospho-S6 (Ser235/236) antibodies were purchased from Cell Signaling Technology; mouse anti-actin and mouse anti-GAPDH was purchased from Abcam and anti-LAMP-1, a marker for LEL, was described earlier^8^. Horseradish peroxidase-conjugated secondary antibodies were purchased from Rockland Immunochemicals, while AlexaFluor secondary antibodies were from Life Technologies.

### Transfection

Cells were transfected with different concentrations of DNA using JetPrime (PolyPlus transfections), according to the manufacturer’s instructions. pcDNA3.1 plasmid was used for Mock transfections. Depletion studies were performed using Lipofectamine 2000 (Life Technologies) to transfect 20 nM of non-silencing siRNA or a pool of siRNA against RagA and RagB (Santa Cruz Biotechnology). 24 h after cells were transfected again with the same amount of siRNA together with pNL4-3 or pcDNA3.1. 24 h after the second round of transfection, cell were fixed or lysed.

### Western blotting

Cells were collected after transfection, washed with DPBS and lysed in ice-cold lysis buffer (adapted from^58^) (50 mM Hepes ph 7.4, 40 mM NaCl, 2 mM EDTA, 1 mM orthovanadate, 50 mM NaF, 10 mM pyrophosphate, 10 mM glycerol phosphate, 1% NP-40, protease and phosphatase inhibitors (Roche)). Cell lysates were quantified by the Bradford assay (Bio-Rad) and 20 µg of lysates were denatured in Laemmli sample buffer and incubated for 5 min at 95 °C. The proteins were separated by SDS-PAGE and transferred onto polyvinylidene difluoride membranes. Membranes were blocked with 5% non-fat milk in Tris-buffered saline and 0.5% Tween 20 (TBST) and then incubated with primary antibodies. After washes with TBST, the membranes were incubated with horseradish peroxidase conjugated secondary antibodies (Rockland Immunochemicals) and detect using Western Lightning Plus-ECL reagent (Perkin-Elmer). Signal intensity was quantified by ImageJ (NIH).

### Cell and parasite culture

HeLa cells were maintained in DMEM (Life Technologies) supplemented with penicillin/streptomycin and 10% fetal bovine serum (Hyclone). THP-1 cell lines were generated by transduction with a VSV-G pseudotype virus consisting of LAIrtTA genome^59, 60^ in which the nucleocapsid portion of Gag had been replaced with a leucine zipper as well as protease and reverse transcriptase domains replaced with GFP in frame with Gag. Following transduction, cells were incubated in 2 μg/mL Doxycycline for several days, then GFP-positive cells isolated by Fluorescence-activated cell sorting (FACS). After expansion of selected cells, subclones were isolated by limiting dilution and screened for GagGFP expression in the presence and absence of doxycycline. THP-1 cells in a monocytic state were differentiated to macrophages for 48 h in RPMI containing 10% FBS and 100 ng/mL phorbol myristate acetate (PMA). Cells were allowed to recover for 48 h in complete RMPI before inducing HIV-1 expression for 72 h with 2 μg/mL Doxycycline. *Leishmania* promastigotes (*L. major* WT and *L. major* GP63^−/−^) were used to infect host cells at a parasite to cell ratio of 20:1 for at least 6 hr as previously described^61^. Effective Leishmania infection and impact on cells was further monitored using anti-gp63 antibody^61^.

### Stress treatment

Cells were serum-starved overnight before each treatment. Cells were treated with 500 µM sodium Ars (NaAsO_2_; MilliporeSigma), 250 nM Torin1, 100 nM Rapamycin, 20 µM LY294002 or 5 µM PI-103 for 1 h, unless otherwise stated. For protein and RNA stability assay cells were treated with 20 µg/mL of Cycloheximide (Sigma), 80 µM 5,6-Dichloro-1-β-D-ribofuranosylbenzimidazole (DRB) (Sigma) and 1 µg/µL Actinomycin D (Sigma) for the indicated time points. For heat shock, cells were incubated for 30 minutes at 42 °C and immediately fixed. For aa and glucose starvation, HeLa cells were washed twice with Dulbecco’s phosphate-buffered saline (DPBS) before being incubated in aa-free DMEM (Wisent) or glucose-free DMEM (Life Technologies) for 1 h. After starvation, recovery was initiated with complete DMEM without serum for 30 minutes before fixation or cell lysis (adapted from refs 7 and 62).

### Fluorescence *in situ* hybridization, immunofluorescence and imaging analyses

FISH/IF co-analyses were performed exactly as described previously^63^. Briefly, after transfection cells were washed once in DPBS and fixed with 4% paraformaldehyde for 20 min. Cells were then washed with PBS, incubated in 0.1 M glycine for 10 min, washed with DPBS, incubated in 0.2% Triton X-100 for 5 min and washed in DPBS. A digoxigenin-labeled RNA probe was synthesized *in vitro* in presence of digoxigenin-labeled UTP (Roche). To stain the vRNA, cells were DNAse (Invitrogen) treated for 15 min (25 U per coverslip), then incubated in hybridization solution for 16–18 h at 42 °C (50% formamide, 1 mg/ml tRNA, 2X SSPE, 5 X Denharts, 5 U RNaseOut (Invitrogen), 50 ng probe). Cells were then incubated in 50% formamide for 15 min at 42 °C and incubated twice in 2 X SSPE for 5 min each at 42 °C. Cells were briefly washed in PBS before being blocked in 1 X blocking solution (Roche). Primary antibodies were applied for 1 h at 37 °C, and then washed for 10 min in PBS followed by secondary antibodies for 1 h. Cells were washed for 20 min in PBS before being mounted on glass slides using ProLong Gold Antifade Reagent with DAPI (Life Technologies). Negative isotype-matched antibody were used to control staining specificity.

Laser confocal microscopy was performed using a Leica DM16000B microscope. The microscope was equipped with a WaveFX spinning disk confocal head (Quorum Technologies), and images were acquired with a Hamamatsu ImageEM EM-charge coupled device camera. Scanning was performed and digitized at a resolution 1,024 × 1,024 pixel. Image processing and analyses were performed by Imaris software v. 8.1.2 (Bitplane/Andor) or by MetaXpress software (Molecular Devices). All imaging experiments were performed at least three times with similar results. The observed phenotypes were representative of n > 100 cells per condition in each experiment.

To quantify S6-p, a single in-focus plane was acquired. Using ImageJ (v1.50b, NIH), an outline was drawn around each cell and the mean fluorescence measured, along with several adjacent background readings. A total of at least 100 cells per conditions (HIV-1 positive or negative), from 3 independent experiments were analyzed.

### Transmission EM

Samples preparation was performed exactly as described in ref. 64. Samples were examined using a transmission electron microscope (H7600; Hitachi) operated at 100 kV. Images were acquired digitally using a 1-megapixel AMT Advantage CCD camera (ORCA; Hamamatsu Photonics).

### Metabolic labeling of newly synthesized Gag

To examine the changes in *de novo* synthesis of Gag upon Torin1 treatment, newly synthesized protein was labeled with Click-iT AHA for Nascent Protein Synthesis kit and Click-iT Biotin Protein Reaction Buffer Kit (Invitrogen) according to manufacturer’s instructions. Briefly, pNL4-3-transfected cells were serum starved overnight. The day after the medium was replaced with methionine-free media containing the Click-IT AHA (L-azidohomoalanine, 50 μM) (Life Technologies) without or with Torin1 and collected at the indicated time points. Cells were lysed with 1% SDS in 50 mM Tris HCl pH 8.0 and the Click-IT reaction was carried out on 200 μg of protein lysate using the Click-IT Protein Reaction Buffer kit (Life Technologies) and biotin-alkyne (40 μM) according to the manufacturer’s instructions. The labeled material was precleared with normal rabbit serum and 25 μL of a 50:50 slurry of protein G-Sepharose (Thermo Scientific), incubated with rabbit anti-p24 (Fitzgerald) for 16 h at 4 °C and with 30 μL of a 50:50 slurry of protein G-Sepharose for 3 h at room temperature.

### Measurement of protein synthesis

Protein synthesis during Torin1 treatment was measured by the incorporation of puromycin into peptide chains as described^65^. Briefly, pNL4-3 and mock transfected HeLa cells were treated up to 4 h with Torin1 and were incubated with 10 μg/ml puromycin (MilliporeSigma) for 10 min before cell lysis. Cell extracts were bloted with anti-Puromycin antibody (12D10, MilliporeSigma) and puromycin incorporation was assessed by summating the immunoblot intensity of all protein bands and subtracting background.

### RT-PCR

RNA was isolated from cell extracts using Aurum Total RNA Mini Kit (BIO-RAD) according to the manufacturer’s protocol. From RNA extracts cDNA was synthesized using the SuperScript IV kit (Life Technologies) and Oligo dT according to the manufacturer’s protocol. 5 μL of cDNA was used along with primers^66^ at 500 nM using 2x GoTaq® reaction mix (Promega) to amplify PCR products in a T Personal Thermocycler (Biometra). PCR products were analyzed by agarose gel electrophoresis.

### Virus purification

Supernatants were collected from pNL4-3 transfected HeLa cells and centrifuged at 1,000 × *g* for 10 minutes at 4 °C to remove cellular debris before being filtered (0.2 μm). Samples were ultracentrifuged at 125,000 × *g* for 1 hour at 4 °C. Virus pellets were resuspended in TNE buffer (10 mM Tris pH 8.0, 100 mM NaCl, 2 mM EDTA) and analyzed by Western blotting.

### Immunoprecipitation (IP) assays

HeLa cells were transfected and then solubilized with NP-40 lysis buffer (50 mM Tris HCl pH 8.0, 150 mM NaCL, 0.5 mM EDTA and 0.5% NP-40). For immunoprecipitation, 500 μg of protein lysate was incubated with 30 μL Dynabeads Protein A (Life Technologies) loaded with anti-RagA, anti-RagB or rabbit IgG isotype control (Abcam) antibodies overnight at 4 °C. Beads were washed with NP40 lysis buffer three times before being eluted with 1X Laemmli sample buffer.

### Statistical analyses

Differences between HIV-1-transfected and mock transfected cells, within the same treatment condition, were tested by unpaired two-tailed Student’s t-test (unless otherwise stated) using the GraphPad v6.1 program (La Jolla, CA, USA). Values of p < 0.05 were considered to be statistically significant.

## Electronic supplementary material

Supplementary information

Supplementary figures

## References

[1] Laplante M, Sabatini DM (2012). mTOR signaling in growth control and disease. Cell.

[2] Yoshida S (2011). Redox regulates mammalian target of rapamycin complex 1 (mTORC1) activity by modulating the TSC1/TSC2-Rheb GTPase pathway. The Journal of biological chemistry.

[3] Kedersha N (2000). Dynamic shuttling of TIA-1 accompanies the recruitment of mRNA to mammalian stress granules. J Cell Biol.

[4] Kedersha NL, Gupta M, Li W, Miller I, Anderson P (1999). RNA-binding proteins TIA-1 and TIAR link the phosphorylation of eIF-2 alpha to the assembly of mammalian stress granules. J Cell Biol.

[5] Takahara T, Maeda T (2012). Transient sequestration of TORC1 into stress granules during heat stress. Molecular cell.

[6] Wippich F (2013). Dual specificity kinase DYRK3 couples stress granule condensation/dissolution to mTORC1 signaling. Cell.

[7] Sancak Y (2008). The Rag GTPases bind raptor and mediate amino acid signaling to mTORC1. Science.

[8] Lehmann M (2009). Intracellular transport of human immunodeficiency virus type 1 genomic RNA and viral production are dependent on dynein motor function and late endosome positioning. The Journal of biological chemistry.

[9] Molle D (2009). Endosomal trafficking of HIV-1 gag and genomic RNAs regulates viral egress. The Journal of biological chemistry.

[10] Nardacci R (2014). Autophagy plays an important role in the containment of HIV-1 in nonprogressor-infected patients. Autophagy.

[11] Heredia A (2015). Targeting of mTOR catalytic site inhibits multiple steps of the HIV-1 lifecycle and suppresses HIV-1 viremia in humanized mice. Proc Natl Acad Sci USA.

[12] Rai P (2013). Rapamycin-induced modulation of HIV gene transcription attenuates progression of HIVAN. Exp Mol Pathol.

[13] Milev MP, Ravichandran M, Khan MF, Schriemer DC, Mouland AJ (2012). Characterization of staufen1 ribonucleoproteins by mass spectrometry and biochemical analyses reveal the presence of diverse host proteins associated with human immunodeficiency virus type 1. Frontiers in microbiology.

[14] Ballou LM, Lin RZ (2008). Rapamycin and mTOR kinase inhibitors. J Chem Biol.

[15] Thoreen CC, Sabatini DM (2009). Rapamycin inhibits mTORC1, but not completely. Autophagy.

[16] Jaramillo M (2011). Leishmania repression of host translation through mTOR cleavage is required for parasite survival and infection. Cell Host Microbe.

[17] Maga JA, Widmer G, LeBowitz JH (1995). Leishmania RNA virus 1-mediated cap-independent translation. Mol Cell Biol.

[18] Korolchuk VI (2011). Lysosomal positioning coordinates cellular nutrient responses. Nature cell biology.

[19] Zoncu R, Efeyan A, Sabatini DM (2011). mTOR: from growth signal integration to cancer, diabetes and ageing. Nature reviews. Molecular cell biology.

[20] Valiente-Echeverria F (2014). eEF2 and Ras-GAP SH3 domain-binding protein (G3BP1) modulate stress granule assembly during HIV-1 infection. Nature communications.

[21] Cinti, A., Le Sage, V., Ghanem, M. & Mouland, A. J. HIV-1 Gag Blocks Selenite-Induced Stress Granule Assembly by Altering the mRNA Cap-Binding Complex. *MBio* **7**, doi:10.1128/mBio.00329-16 (2016). 10.1128/mBio.00329-16 PMC4817256 27025252

[22] Menon S (2014). Spatial control of the TSC complex integrates insulin and nutrient regulation of mTORC1 at the lysosome. Cell.

[23] Sancak Y (2010). Ragulator-Rag complex targets mTORC1 to the lysosomal surface and is necessary for its activation by amino acids. Cell.

[24] Farny NG, Kedersha NL, Silver PA (2009). Metazoan stress granule assembly is mediated by P-eIF2alpha-dependent and -independent mechanisms. Rna.

[25] Rocha N (2009). Cholesterol sensor ORP1L contacts the ER protein VAP to control Rab7-RILP-p150 Glued and late endosome positioning. J Cell Biol.

[26] Li X (2016). A molecular mechanism to regulate lysosome motility for lysosome positioning and tubulation. Nature cell biology.

[27] Kumar A, Herbein G (2014). The macrophage: a therapeutic target in HIV-1 infection. Mol Cell Ther.

[28] McNulty S, Flint M, Nichol ST, Spiropoulou CF (2013). Host mTORC1 signaling regulates andes virus replication. J Virol.

[29] Gerber PP (2015). Rab27a controls HIV-1 assembly by regulating plasma membrane levels of phosphatidylinositol 4,5-bisphosphate. J Cell Biol.

[30] Milev MP, Brown CM, Mouland AJ (2010). Live cell visualization of the interactions between HIV-1 Gag and the cellular RNA-binding protein Staufen1. Retrovirology.

[31] Blanchet FP (2010). Human immunodeficiency virus-1 inhibition of immunoamphisomes in dendritic cells impairs early innate and adaptive immune responses. Immunity.

[32] Sagnier S (2015). Autophagy restricts HIV-1 infection by selectively degrading Tat in CD4+ T lymphocytes. J Virol.

[33] Brasey A (2003). The leader of human immunodeficiency virus type 1 genomic RNA harbors an internal ribosome entry segment that is active during the G2/M phase of the cell cycle. J Virol.

[34] Monette A (2013). Dual mechanisms of translation initiation of the full-length HIV-1 mRNA contribute to gag synthesis. PLoS One.

[35] Ramirez-Valle F, Badura ML, Braunstein S, Narasimhan M, Schneider RJ (2010). Mitotic raptor promotes mTORC1 activity, G(2)/M cell cycle progression, and internal ribosome entry site-mediated mRNA translation. Mol Cell Biol.

[36] Di Benedetto F (2010). First report on a series of HIV patients undergoing rapamycin monotherapy after liver transplantation. Transplantation.

[37] Li X (2011). Mammalian target of rapamycin inhibition in macrophages of asymptomatic HIV+ persons reverses the decrease in TLR-4-mediated TNF-alpha release through prolongation of MAPK pathway activation. J Immunol.

[38] von Boehmer H, Daniel C (2013). Therapeutic opportunities for manipulating T(Reg) cells in autoimmunity and cancer. Nat Rev Drug Discov.

[39] Besnard E (2016). The mTOR Complex Controls HIV Latency. Cell Host Microbe.

[40] Martin AR (2017). Rapamycin-mediated mTOR inhibition uncouples HIV-1 latency reversal from cytokine-associated toxicity. J Clin Invest.

[41] Coleman EM, Walker TN, Hildreth JE (2012). Loss of Niemann Pick type C proteins 1 and 2 greatly enhances HIV infectivity and is associated with accumulation of HIV Gag and cholesterol in late endosomes/lysosomes. Virol J.

[42] Tang Y, Leao IC, Coleman EM, Broughton RS, Hildreth JE (2009). Deficiency of niemann-pick type C-1 protein impairs release of human immunodeficiency virus type 1 and results in Gag accumulation in late endosomal/lysosomal compartments. J Virol.

[43] Caillet M (2011). Rab7A is required for efficient production of infectious HIV-1. PLoS Pathog.

[44] Arainga M (2015). Opposing regulation of endolysosomal pathways by long-acting nanoformulated antiretroviral therapy and HIV-1 in human macrophages. Retrovirology.

[45] Syed F, McCrae MA (2009). Interactions *in vivo* between the Vif protein of HIV-1 and the precursor (Pr55(GAG)) of the virion nucleocapsid proteins. Arch Virol.

[46] Sheehy AM, Gaddis NC, Choi JD, Malim MH (2002). Isolation of a human gene that inhibits HIV-1 infection and is suppressed by the viral Vif protein. Nature.

[47] Shen K, Sidik H, Talbot WS (2016). The Rag-Ragulator Complex Regulates Lysosome Function and Phagocytic Flux in Microglia. Cell Rep.

[48] Kim YC (2014). Rag GTPases are cardioprotective by regulating lysosomal function. Nature communications.

[49] Johnson DE, Ostrowski P, Jaumouille V, Grinstein S (2016). The position of lysosomes within the cell determines their luminal pH. J Cell Biol.

[50] Hurtado-Lorenzo A (2006). V-ATPase interacts with ARNO and Arf6 in early endosomes and regulates the protein degradative pathway. Nature cell biology.

[51] Abrahamyan LG (2010). Novel Staufen1 ribonucleoproteins prevent formation of stress granules but favour encapsidation of HIV-1 genomic RNA. Journal of cell science.

[52] Settembre C (2012). A lysosome-to-nucleus signalling mechanism senses and regulates the lysosome via mTOR and TFEB. EMBO J.

[53] Le Sage, V., Cinti, A., Amorim, R. & Mouland, A. J. Adapting the Stress Response: Viral Subversion of the mTOR Signaling Pathway. *Viruses* **8**, doi:10.3390/v8060152 (2016). 10.3390/v8060152 PMC4926172 27231932

[54] Karczewski MK, Strebel K (1996). Cytoskeleton association and virion incorporation of the human immunodeficiency virus type 1 Vif protein. J Virol.

[55] Lingappa JR, Dooher JE, Newman MA, Kiser PK, Klein KC (2006). Basic residues in the nucleocapsid domain of Gag are required for interaction of HIV-1 gag with ABCE1 (HP68), a cellular protein important for HIV-1 capsid assembly. The Journal of biological chemistry.

[56] Poon DT, Chertova EN, Ott DE (2002). Human immunodeficiency virus type 1 preferentially encapsidates genomic RNAs that encode Pr55(Gag): functional linkage between translation and RNA packaging. Virology.

[57] van der Kant R (2013). Late endosomal transport and tethering are coupled processes controlled by RILP and the cholesterol sensor ORP1L. Journal of cell science.

[58] Sarbassov DD, Guertin DA, Ali SM, Sabatini DM (2005). Phosphorylation and regulation of Akt/PKB by the rictor-mTOR complex. Science.

[59] Zhou X (2006). The genetic stability of a conditional live HIV-1 variant can be improved by mutations in the Tet-On regulatory system that restrain evolution. The Journal of biological chemistry.

[60] Zhou X, Vink M, Berkhout B, Das AT (2006). Modification of the Tet-On regulatory system prevents the conditional-live HIV-1 variant from losing doxycycline-control. Retrovirology.

[61] Isnard A (2015). Impact of Leishmania infection on host macrophage nuclear physiology and nucleopore complex integrity. PLoS Pathog.

[62] Gao M (2014). Site-specific activation of AKT protects cells from death induced by glucose deprivation. Oncogene.

[63] Vyboh, K., Ajamian, L. & Mouland, A. J. Detection of viral RNA by fluorescence *in situ* hybridization (FISH). *J Vis Exp*, e4002, doi:10.3791/4002 (2012). 10.3791/4002 PMC3466955 22588480

[64] Monette A, Pante N, Mouland AJ (2011). HIV-1 remodels the nuclear pore complex. J Cell Biol.

[65] Schmidt EK, Clavarino G, Ceppi M, Pierre P (2009). SUnSET, a nonradioactive method to monitor protein synthesis. Nat Methods.

[66] Beriault V (2004). A late role for the association of hnRNP A2 with the HIV-1 hnRNP A2 response elements in genomic RNA, Gag, and Vpr localization. The Journal of biological chemistry.

