# Supplementary material for: HIV-1 enhances mTORC1 activity and repositions lysosomes to the periphery by co-opting Rag GTPases
Source: Sci Rep. 2017 Jul 14;7:5515. doi: 10.1038/s41598-017-05410-0 (PMC5511174; doi:10.1038/s41598-017-05410-0)
Supplement: Supplementary file 2 — Supplementary figures [file 41598_2017_5410_MOESM2_ESM.pdf]

Supplementary Information

HIV-1 enhances mTORC1 activity and repositions lysosomes to the periphery by co-opting Rag GTPases

Alessandro Cinti[1,2], Valerie Le Sage[1*], Miroslav P. Milev[1,2*], Fernando Valiente-Echeverría[1,2,3], Christina Crossie[1,2], Marie-Joelle Miron[1], Nelly Panté[4], Martin Olivier[2,5] and Andrew J. Mouland[1,2,5]

[1]HIV-1 RNA Trafficking Laboratory, Lady Davis Institute at the Jewish General Hospital, Montréal, Québec, Canada H3T 1E2

[2]Department of Medicine and the Division of Experimental Medicine, McGill University, Montréal, Québec, Canada H3A 0G4

[3]Current address: Molecular and Cellular Virology Laboratory, Virology Program, Institute of Biomedical Sciences, Faculty of Medicine, Universidad de Chile, Independencia 834100, Santiago, Chile

[4]Department of Zoology, University of British Columbia, Vancouver, British Columbia, Canada V6T 1Z4

[5]Department of Microbiology and Immunology, McGill University, Montréal, Québec, Canada H3A 2B4

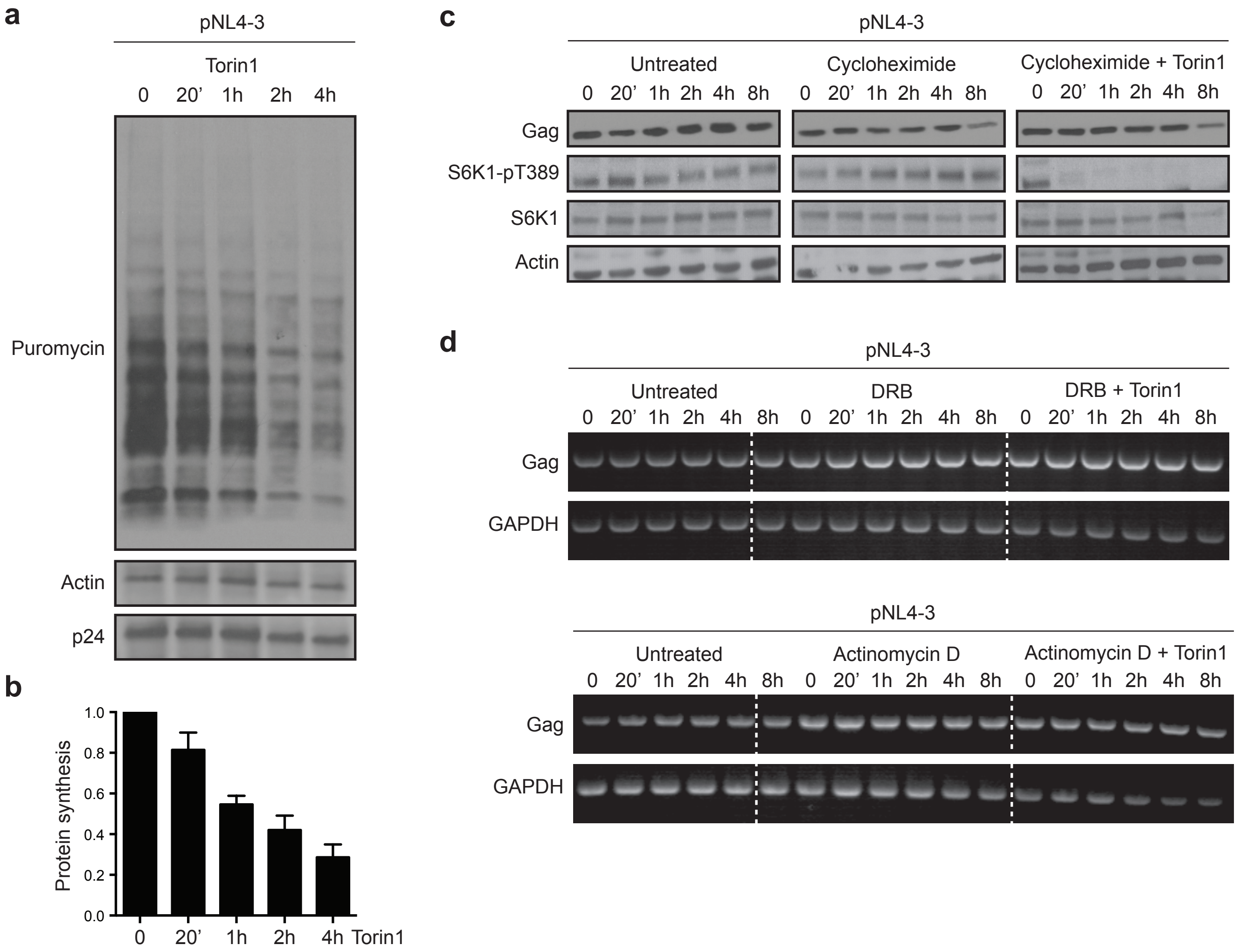

Supplementary Figure 1. Torin1 treatment decreased protein synthesis as measured *in vitro* with puromycin-labeling technique and do not stabilize Gag-protein and RNA. Measurements of protein synthesis were performed by incubating pNL4-3-transfected HeLa cells with medium containing puromycin as described in Materials and Methods. (**a**) HeLa extracts were separated by denaturing electrophoresis and analyzed by western blot with antibody to puromycin (12D10). Actin immunoblot is shown as a loading control (bottom). (**b**) Quantification of the puromycin-labeled peptides, values were normalized against untreated (time 0) cells extracts. (**c**) pNL4-3-transfected HeLa cells were incubated with Cycloheximide alone or in combination with Torin1 and HIV-1 Gag stability was assesed by western blot during the indicated time points. (**d**) pNL4-3-transfected HeLa cells were incubated with DRB or Actinomycin D alone or in combination with Torin1 and HIV-1 Gag-RNA stability was assesed by RT-PCR during the indicated time points.

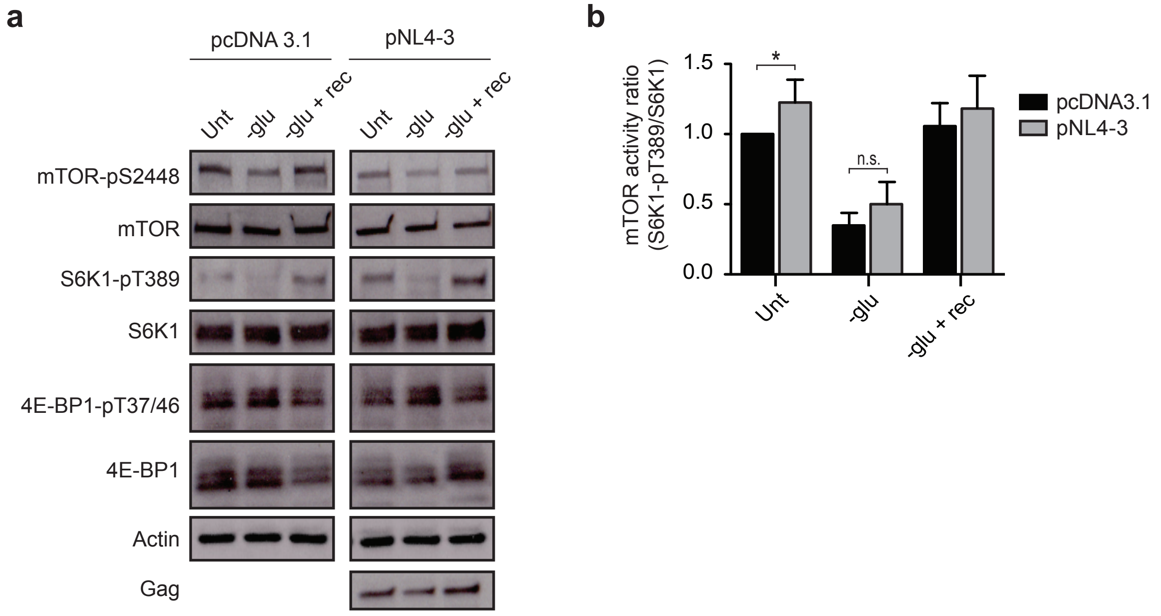

Supplementary Figure 2. HIV-1 does not maintain mTORC1 activation during glucose starvation. (**a**) HeLa cells were transfected with either pcDNA3.1 or pNL4-3 and starved for glucose (glu) (and serum) for 1 h and subsequently recovered (rec) with complete media (serum free) for an additional 30 minutes. Cell lysates were subjected to SDS-PAGE, immunoblotted and probed with the indicated antibodies. (**b**) The graphs show the ratio of S6K1-pT389/total S6K1 for mock or HIV-1-transfected cells as in a. Values were normalized against untreated (Unt) cells transfected with pcDNA3.1. The results are presented as the mean ± S.D. from three different experiments. P value is indicated by * ($p < 0.05$); n.s.: no signifcant difference between the means.

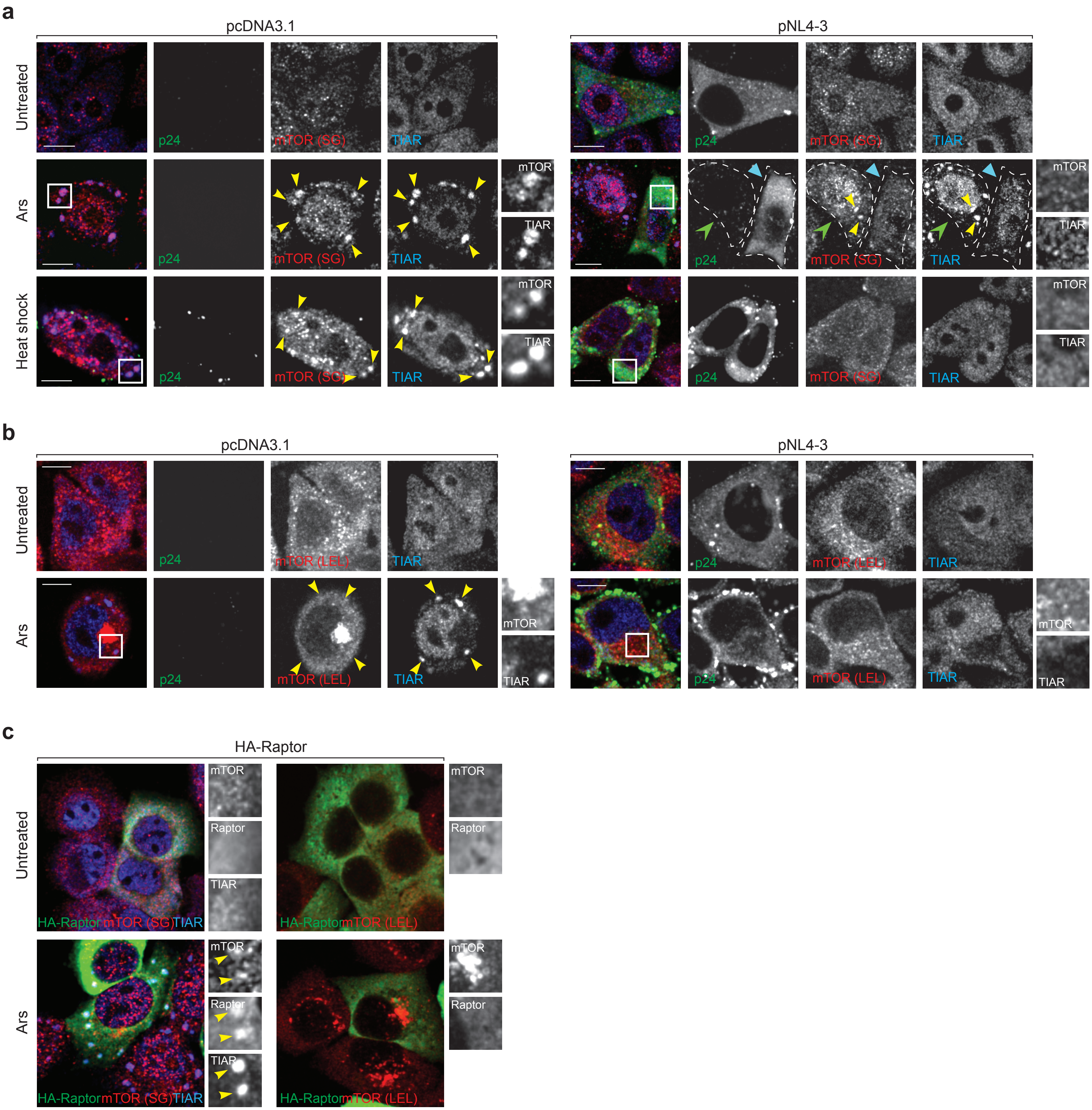

Supplementary Figure 3. HIV-1 alters the localization of the SG and LEL-associated mTOR populations. HeLa cells were transfected with pcDNA3.1 or pNL4-3 and either stressed using 500 nM Ars for 50 min or heat stressed at 42 °C for 1h. (**a**) Cells were stained for the stress granule (SG) pool of mTOR (identified using Santa-Cruz, antibody sc-1549), p24 and the SG marker TIAR. Yellow arrowheads identify SGs. Light blue arrowheads identify HIV-1-expressing cells, while HIV-1-negative cells in the same field are indicated with green arrowheads. (**b**) The lysosomal-LEL subpopulation of mTOR (identified using Cell Signaling, antibody 7C10), p24 and TIAR were stained. Yellow arrowheads identify sGs. The results shown are representative of two experiments. (c) HeLa cells were transfected with HA-Raptor and stressed using 500 nM Ars for 50 min. Cells were stained for the SG or LEL pool of mTOR, HA and the SG marker TIAR. Yellow arrowheads identify SGs. Scale bars are 10 μm.

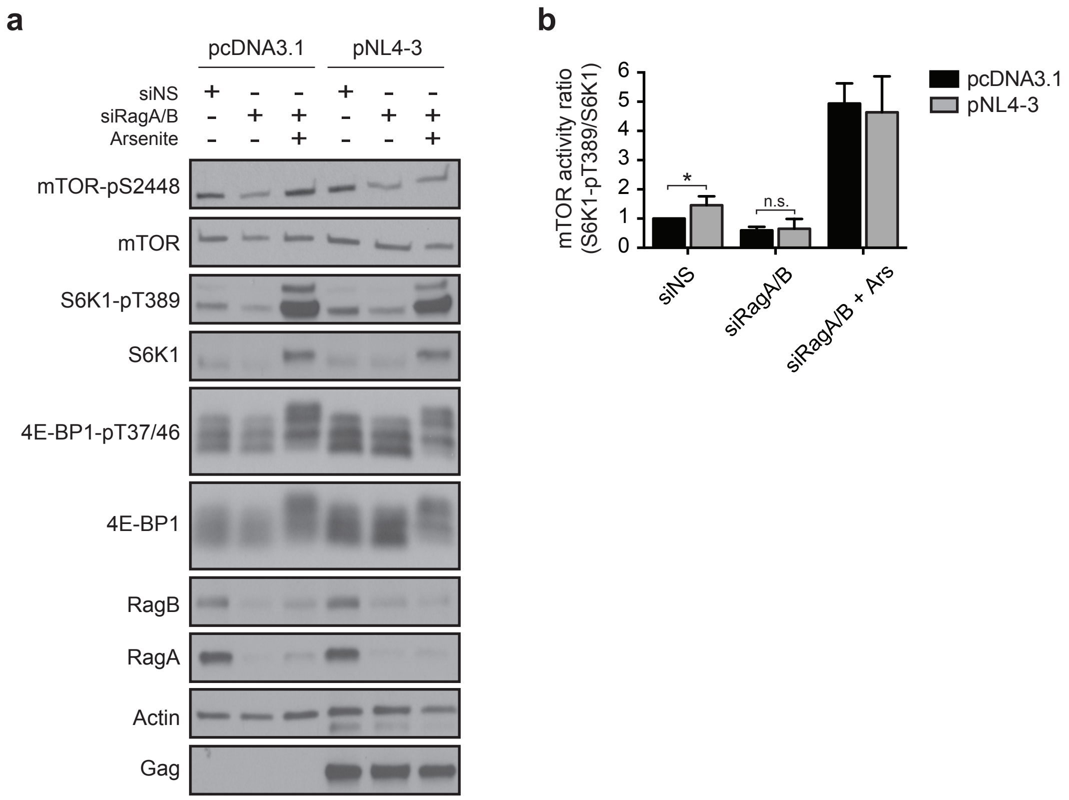

Supplementary Figure 4. HIV-1 does not modulate mTOR activity upon depletion of RagA and RagB. (**a**) Lysates from pcDNA3.1- or pNL4-3-transfected HeLa cells were subjected to immunoprecipitation with RagA antibodies. Samples were subjected to SDS-PAGE followed by transfer to nitrocellulose membrane and probed with the indicated antibodies. (**b**) The graphs show the ratio of S6K1-pT389/total S6K1 for siRagA/B-transfected cells versus those in control siNS-transfected cells. Values were normalized against untreated cells transfected with pcDNA3.1 and are presented as the mean ± S.D. from three different experiments. P value is indicated by * ($p < 0.05$).

Fig.1

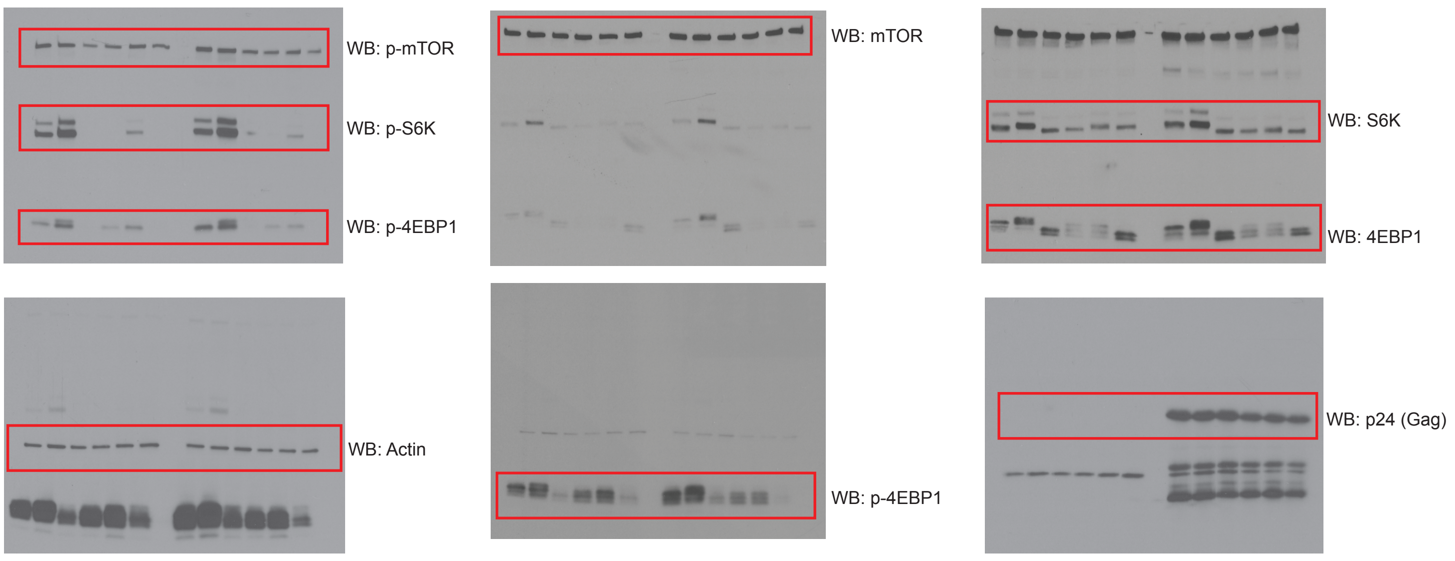

Fig. 2

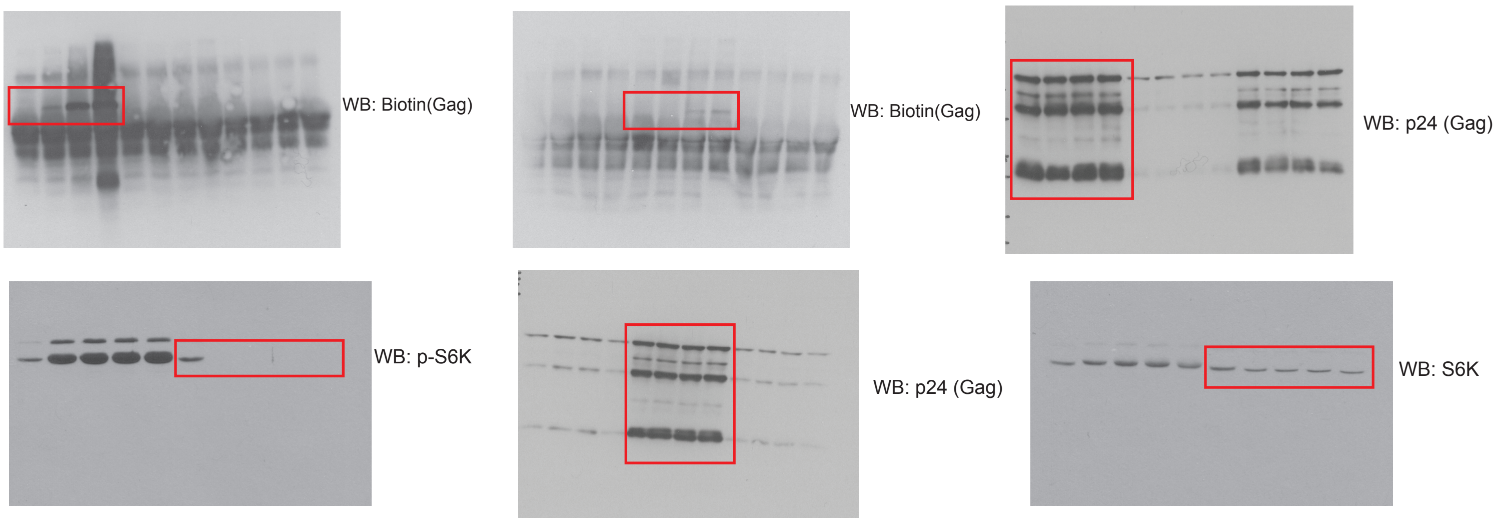

Fig. 3

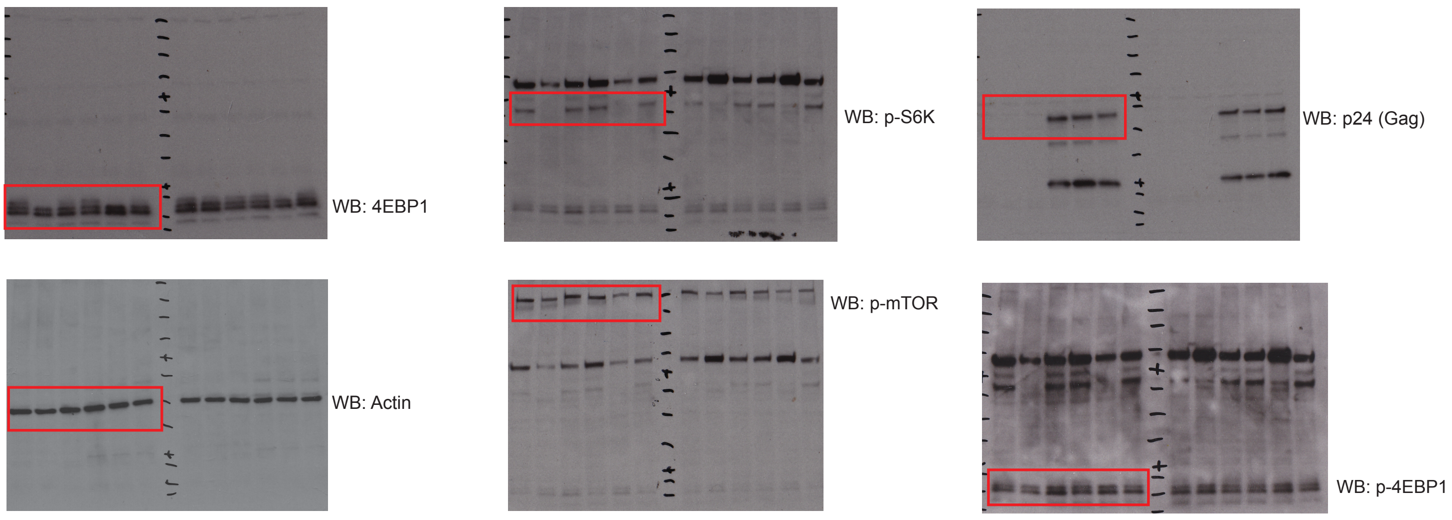

Supplementary Figure 5. Full scans of most important western blots with indicated areas of selection

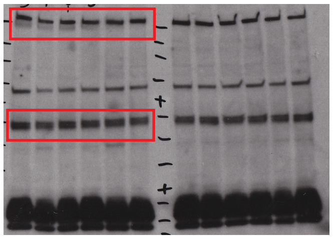

WB: mTOR

WB: p-S6K

Fig. 5

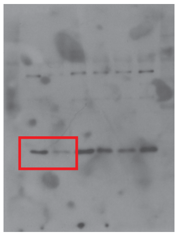

WB: p24

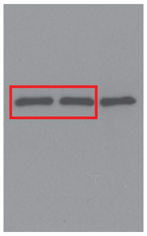

WB: GAPDH

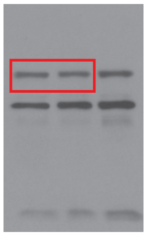

WB: p24 (Gag)

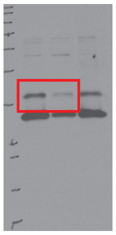

WB: RagB

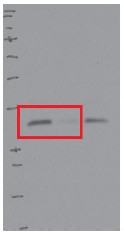

WB: RagA

Fig. 6

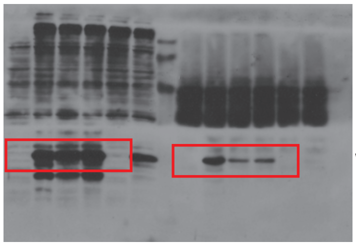

WB: Vif

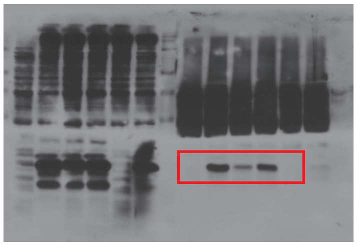

WB: Vif

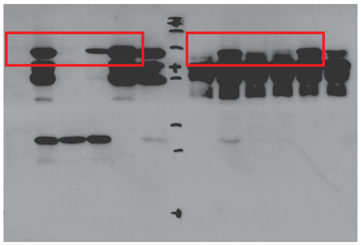

WB: p24 (Gag)

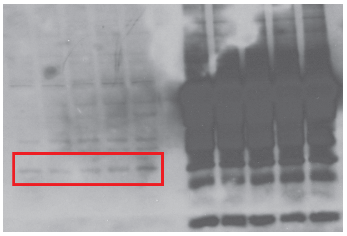

WB: RagA

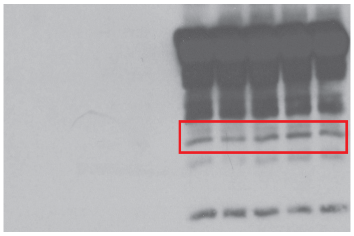

WB: RagA

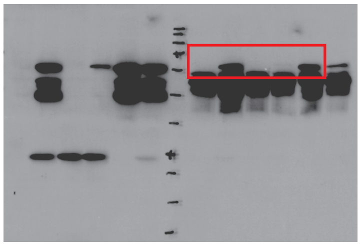

WB: p24 (Gag)

Supplementary Figure 5 continued

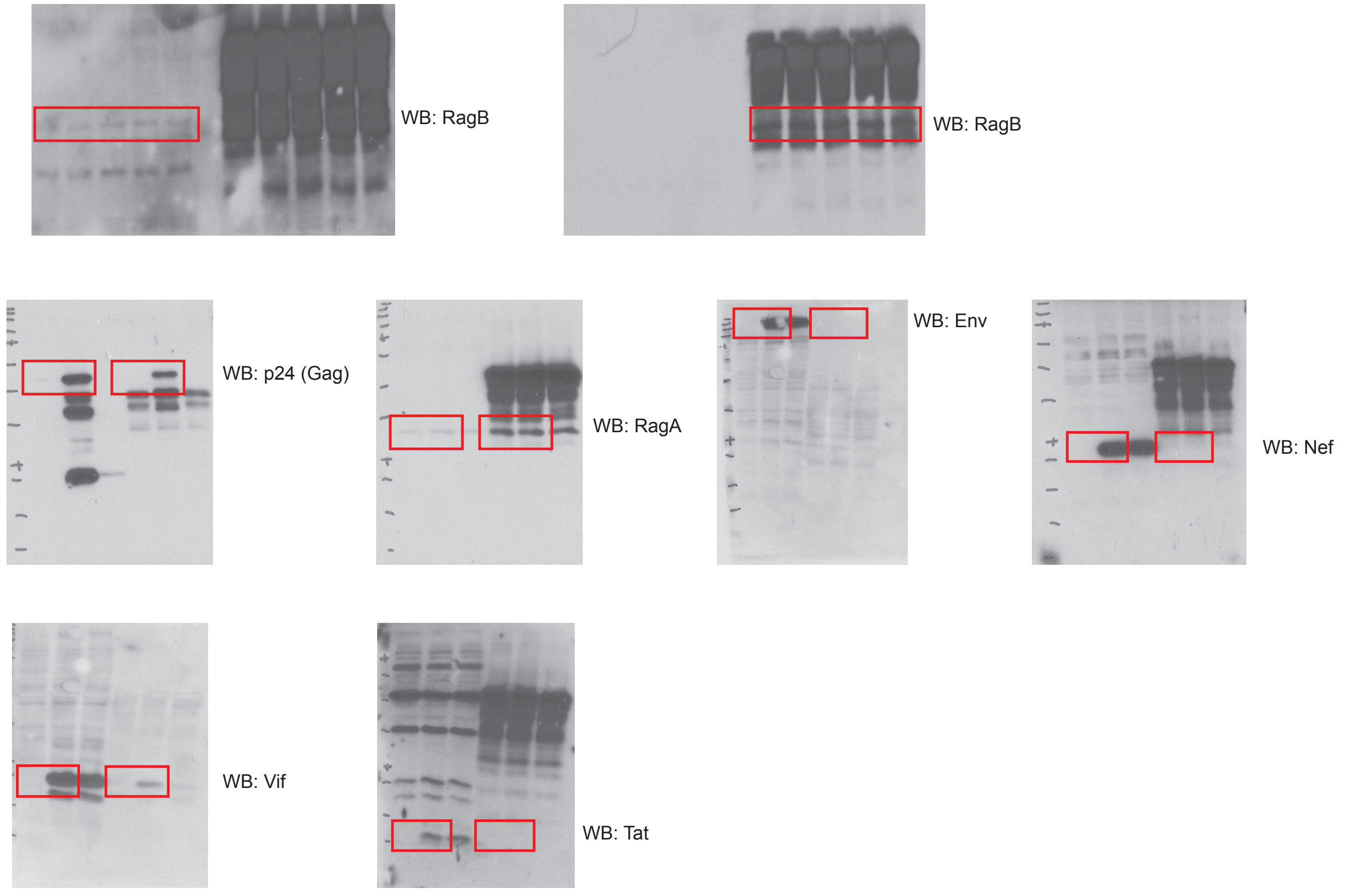

Supp. Fig. 2

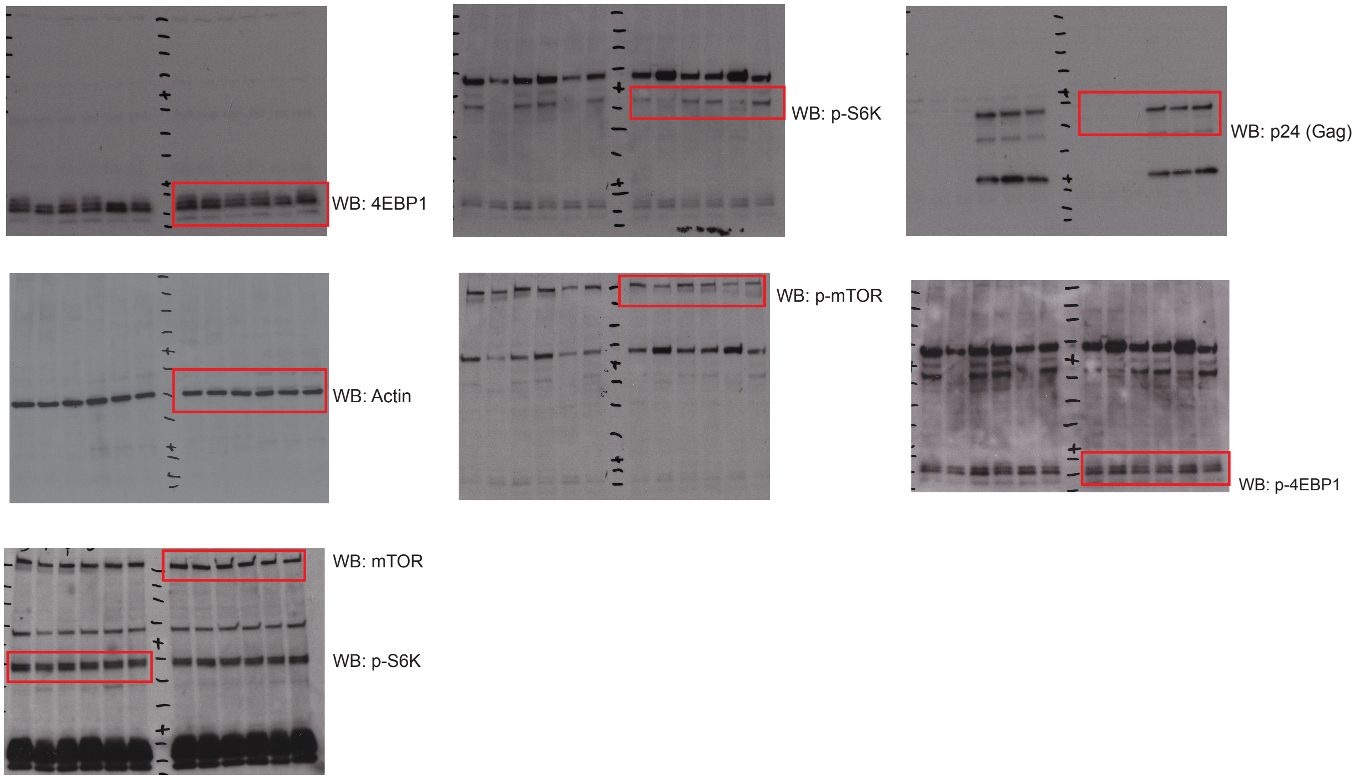

Supplementary Figure 5 continued

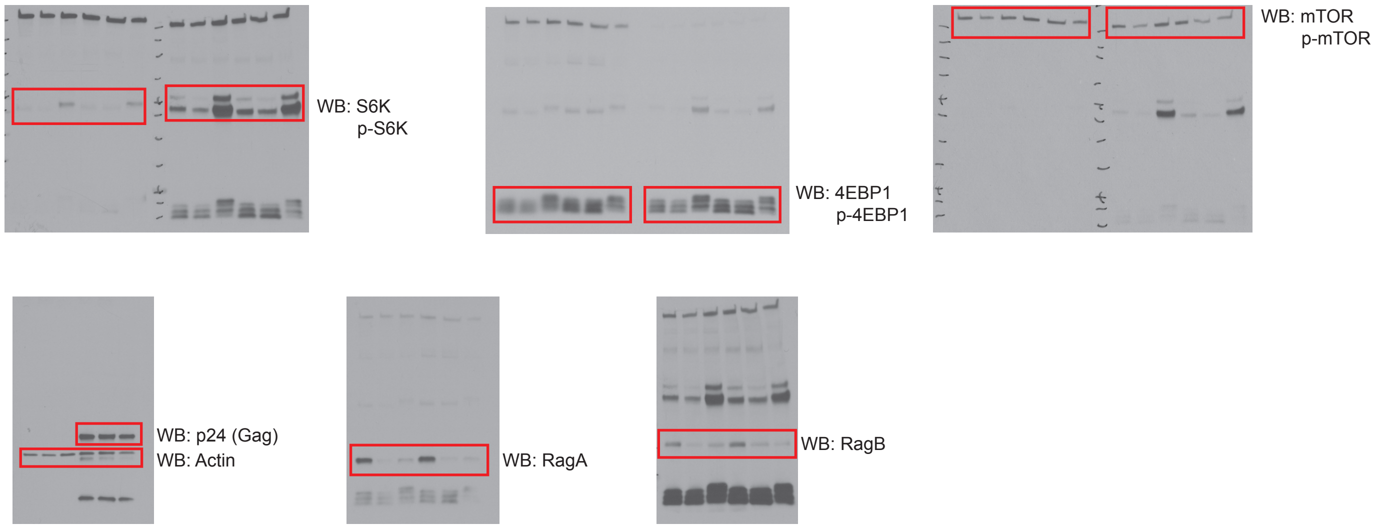

Supplementary Figure 5 continued

Supplementary Movie 1. HIV-1 blocks LEL clustering induced by Arsenite. HeLa cells were transfected with LAMP1-GFP and pNL4-3/GagmRFP and 24 h after stressed with 500 μM Arsenite for 1 h. Images were collected from minute 0 to minute 60 of Ars treatment, at 1 min intervals in LCM.
